# Scoulerine affects microtubule structure, inhibits proliferation, arrests cell cycle and thus culminates in the apoptotic death of cancer cells

**DOI:** 10.1038/s41598-018-22862-0

**Published:** 2018-03-19

**Authors:** Klara Habartova, Radim Havelek, Martina Seifrtova, Karel Kralovec, Lucie Cahlikova, Jakub Chlebek, Eva Cermakova, Nadezda Mazankova, Jana Marikova, Jiri Kunes, Lucie Novakova, Martina Rezacova

**Affiliations:** 1 Department of Medical Biochemistry, Faculty of Medicine in Hradec Kralove, Charles University, Simkova 870, Hradec Kralove, 500 03 Czech Republic; 2 Department of Biological and Biochemical Sciences, Faculty of Chemical Technology, University of Pardubice, Studentska 573, Pardubice, 532 10 Czech Republic; 3 ADINACO Research group, Department of Pharmaceutical Botany and Ecology, Faculty of Pharmacy, Charles University, Heyrovskeho 1203, Hradec Kralove, 500 05 Czech Republic; 4 Department of Medical Biophysics, Faculty of Medicine in Hradec Kralove, Charles University, Simkova 870, Hradec Kralove, 500 03 Czech Republic; 5 Department of Organic and Bioorganic Chemistry, Faculty of Pharmacy, Charles University, Heyrovskeho 1203, Hradec Kralove, 500 05 Czech Republic; 6 Department of Analytical Chemistry, Faculty of Pharmacy, Charles University, Heyrovskeho 1203, Hradec Kralove, 500 05 Czech Republic

## Abstract

Scoulerine is an isoquinoline alkaloid, which indicated promising suppression of cancer cells growth. However, the mode of action (MOA) remained unclear. Cytotoxic and antiproliferative properties were determined in this study. Scoulerine reduces the mitochondrial dehydrogenases activity of the evaluated leukemic cells with IC_50_ values ranging from 2.7 to 6.5 µM. The xCELLigence system revealed that scoulerine exerted potent antiproliferative activity in lung, ovarian and breast carcinoma cell lines. Jurkat and MOLT-4 leukemic cells treated with scoulerine were decreased in proliferation and viability. Scoulerine acted to inhibit proliferation through inducing G2 or M-phase cell cycle arrest, which correlates well with the observed breakdown of the microtubule network, increased Chk1 Ser345, Chk2 Thr68 and mitotic H3 Ser10 phosphorylation. Scoulerine was able to activate apoptosis, as determined by p53 upregulation, increase caspase activity, Annexin V and TUNEL labeling. Results highlight the potent antiproliferative and proapoptotic function of scoulerine in cancer cells caused by its ability to interfere with the microtubule elements of the cytoskeleton, checkpoint kinase signaling and p53 proteins. This is the first study of the mechanism of scoulerine at cellular and molecular level. Scoulerine is a potent antimitotic compound and that it merits further investigation as an anticancer drug.

## Introduction

**Figure 1:**
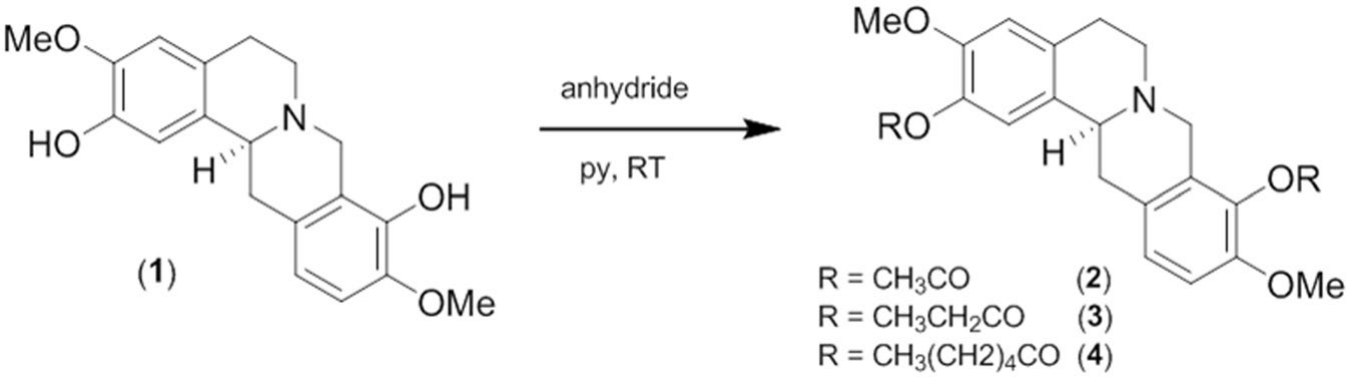
Chemical structure and reaction scheme for acylation of scoulerine (**1**) to 2,9-di-*O*-acetylscoulerine (**2**); 2,9-di-*O*-propionylscoulerine (**3**) and 2,9-di-*O*-hexanoylscoulerine (**4**).

Plant natural compounds and their derivatives continue to provide an indispensable source of new drug leads for drug development. In the area of cancer therapy, up to 80% of approved drugs are either natural products per se or are based thereon^1^. Natural isoquinoline alkaloids as contained in plant extract remedies have been used in traditional medicine for centuries (e.g. Hippocrates of Cos, Pliny the Elder) and have wide-ranging properties that play an important role in the human combat against diseases. Strangely, although various plant families have been extensively investigated in search for constituents with a therapeutic significance, the alkaloids found in the Papaveraceae family plants have not been well studied so far. Among the Papaveraceae alkaloids which are known to possess some bioactive properties, scoulerine (**1**) (Fig. 1) stimulated our investigation. Protoberberine alkaloid scoulerine, also known as discretamine and aequaline, can be found in *Croton flavens*^2^, *Corydalis dubia*^3^ and *Corydalis cava*^4,5^. Scoulerine is an important intermediate in the isoquinoline alkaloids biosynthesis. Scoulerine is made from reticuline and serves as the precursor in the biosynthetic pathway for berberine, stylopine, protopine and sanginarine^6,7^. An initial biological study described its significant *in vitro* antiplasmodial activity against the *P. falciparum* strains, TM4/8.2 (a wild type chloroquine and antifolate sensitive strain) and K1CB1 (multidrug resistant strain), with IC_50_ values 1.78 µg/mL and 1.04 µg/mL, respectively. Regrettably, this activity does not meet the criteria stipulated under the Medicines for Malaria Venture^3^. Other research efforts, performed on rats, determined that scoulerine protects α-adrenoreceptors against irreversible blockade by phenoxybenzamine, inhibits [^3^H]-inositol monophosphate formation caused by noradrenaline^8^ and acts as a selective α_1D_-adrenoreceptor antagonist without affecting the contraction of the rat aorta^9^. Scoulerine has also been reported to exhibit other useful pharmacological properties such as antiemetic, antitussive and antibacterial action^3^ and has been found to have an affinity to the GABA receptors^2^. Interestingly, a pioneer cell culture study on this alkaloid described that scoulerine shows significant cytotoxic activity against A549 and HT-29 cancer cell lines. The authors imply that the cytotoxic potency of scoulerine is associated with its ability to stabilize the covalent topoisomerase I - DNA complex to promote the formation of single-strand DNA breaks^10^. It should be pointed out that the unique position of scoulerine in backbone arrangements during biosynthesis and its interesting biological activities already attracted our attention in two previous studies. Scoulerine was found to be active as an inhibitor of ß-site amyloid precursor protein cleaving enzyme 1 (BACE1), which is a very promising target for the treatment of Alzheimer’s disease (AD)^5^. In our follow-up work, when considering forty-six isoquinoline alkaloids screened by MTT assay, scoulerine exhibited impressive cytostatic activity against gastrointestinal cancer cells^11^. Although our recent study demonstrated the bioactivity of scoulerine with an emphasis on the cytostatic action that may be of interest in cancer chemotherapy, further studies remain to be undertaken to better explore its anticancer potential. At present, this study provides a better investigation of the MOA of scoulerine at cellular and molecular level. In addition to that, the pro-apoptotic and cell cycle arrest activity in p53-deficient (Jurkat) and p53 wild-type (MOLT-4) leukemic cells following scoulerine treatment is determined. Finally, aiming at the further conceptual extension to study structure-cytotoxicity relationships, we have introduced three (**2**, **3** and **4**) aliphatic derivates of scoulerine incorporating esters of carboxylic acids.

## Results

### Scoulerine decreases proliferation of human cancer cells

**Figure 2:**
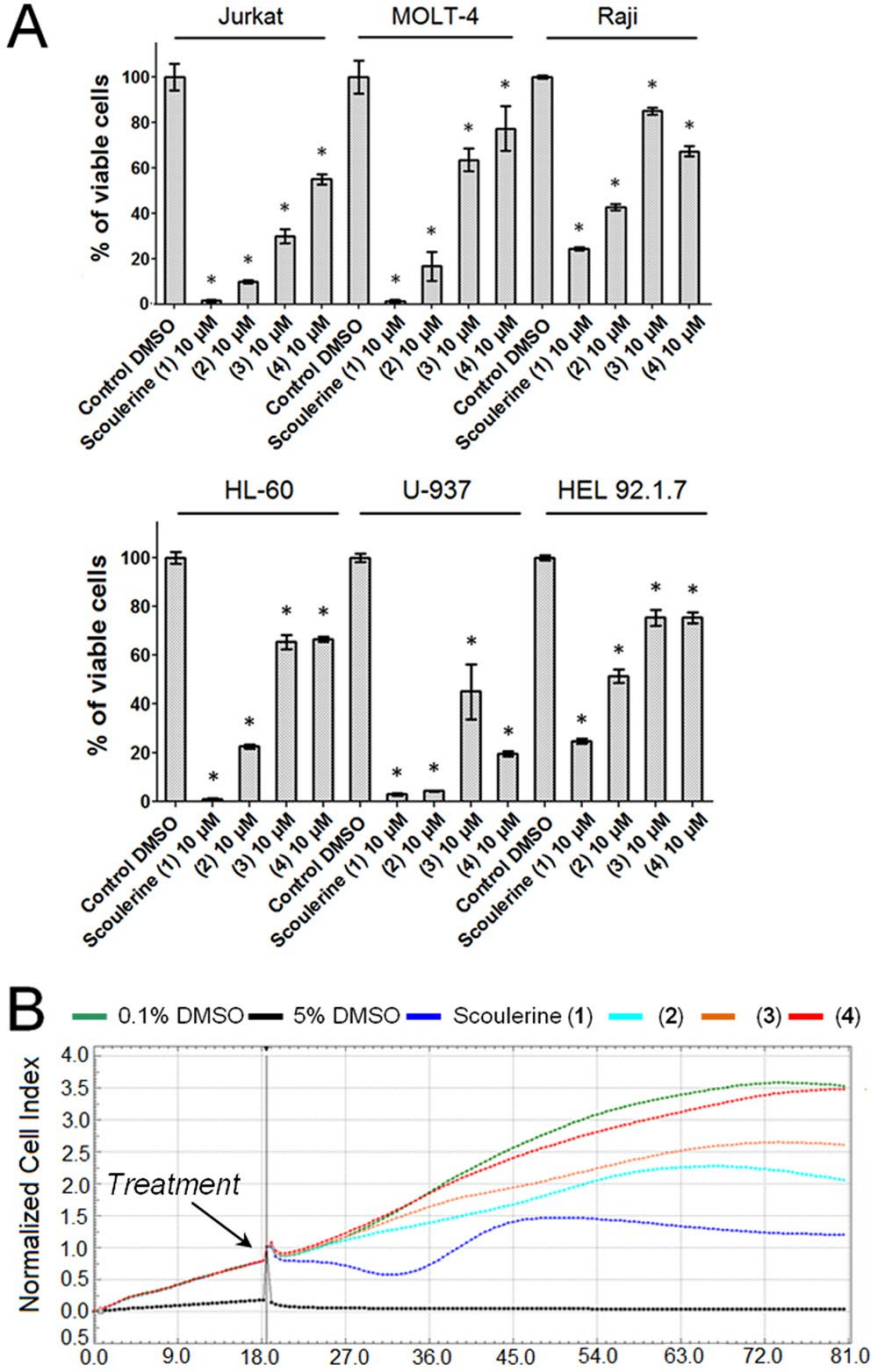
Cytotoxicity of scoulerine (**1**), (**2**), (**3**) and (**4**) following a single-dose exposure at a concentration of 10 µM. Cell proliferation and viability of Jurkat, MOLT-4, Raji, HL-60, U-937 and HEL 92.1.7 cells measured by using XTT assay 48 h after treatment. Viability is referred to cells treated with 0.1% DMSO (Control DMSO). Data are shown as mean values ± SD of at least three independent experiments. *Significantly different to control (P ≤ 0.05) (**A**). Dynamic real-time xCELLigence screen of proliferation and cytotoxicity over 62 h. The human A2780 ovarian carcinoma cells in the logarithmic growth phase were treated. The negative control cells were exposed to 0.1% DMSO (vehicle) and 5% DMSO was used as a positive control. The plot is representative of at least three experiments performed (**B**).

**Figure 3:**
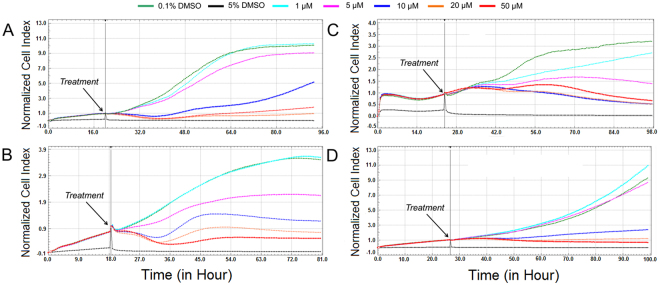
Dynamic real-time monitoring of proliferation and cytotoxicity using the xCELLigence system dedicated to adherent cell lines. Growth kinetics of human A549 lung carcinoma (**A**), A2780 ovarian carcinoma (**B**), SK-BR-3 breast adenocarcinoma (**C**) and MCF-7 breast adenocarcinoma (**D**) cells treated with scoulerine. Cells treated with 0.1% DMSO were used as vehicle control and 5% DMSO treated cells were used as positive control. The normalized cell index was measured over 72 h. Plots shown are representative of at least three replicate experiments in each case.

Various leukemic cell lines and carcinoma cell lines obtained from solid tumors were used for evaluating their sensitivity towards scoulerine. First, scoulerine (**1**) and its three semi-synthetic derivatives (**2**), (**3**) and (**4**) were evaluated at a single-dose exposure (10 µM) against a mini-panel of leukemic cell lines (MOLT-4, Jurkat, Raji, HL-60, U-937 and HEL 92.1.7) using the XTT metabolic activity assay (Fig. 2A) and A2780 ovarian carcinoma cells using the xCELLigence system (Fig. 2B). In order to determine the IC_50_ values, a broad concentration range of scoulerine were determined (Supplementary Fig. 1). Considering the effect of aliphatic esters of scoulerine with varying carbon chain lengths, parent scoulerine proved to be the most active compound against the determined cell lines, with the IC_50_ value ranging from 2.7 µM to 6.5 µM in human leukemic cells (Table 1). Further testing of the scoulerine effects on viability and proliferation revealed that in concentrations of 2.5, 5, 10, 15 and 20 µM scoulerine significantly reduced the viability and proliferation of Jurkat and MOLT-4 cells in a dose dependent manner within 24 h of treatment. Moreover, the reduction of cell viability was even more pronounced 48 h after scoulerine application (Supplementary Fig. 2). Similar results were achieved using the dynamic real-time monitoring of proliferation by the xCELLigence system designated for adherent cell lines. In these experiments, the xCELLigence system was utilized to follow the proliferation of scoulerine and sham-treated cells over a 72 h period. As shown in Fig. 3, scoulerine (10, 20 and 50 µM) had a clear negative impact on human lung carcinoma (A549), ovarian carcinoma (A2780) and breast adenocarcinoma (SK-BR-3 and MCF-7) proliferation. Cells treated with lower doses (1 and 5 µM) of scoulerine experienced unaffected or slightly delayed proliferation.

**Table 1:** IC_50_ values of the scoulerine against mini-panel of human leukemic cells with different tumor suppressor protein p53 gene (TP53) status^a,b^.

Cell type	TP53 status	Scoulerine
MOLT-4	wild-type	4.7 ± 0.2
Jurkat	mutated	2.7 ± 0.1
Raji	mutated	6.5 ± 0.7
HL-60	null	4.2 ± 0.2
U-937	mutated	4.8 ± 0.3
HEL 92.1.7	wild-type	6.3 ± 0.8

^a^Results are expressed in µM.

^b^Results are the mean values ± standard deviations of at least three independent replications.

### Scoulerine induces MOLT-4 and Jurkat cells apoptosis

**Figure 4:**
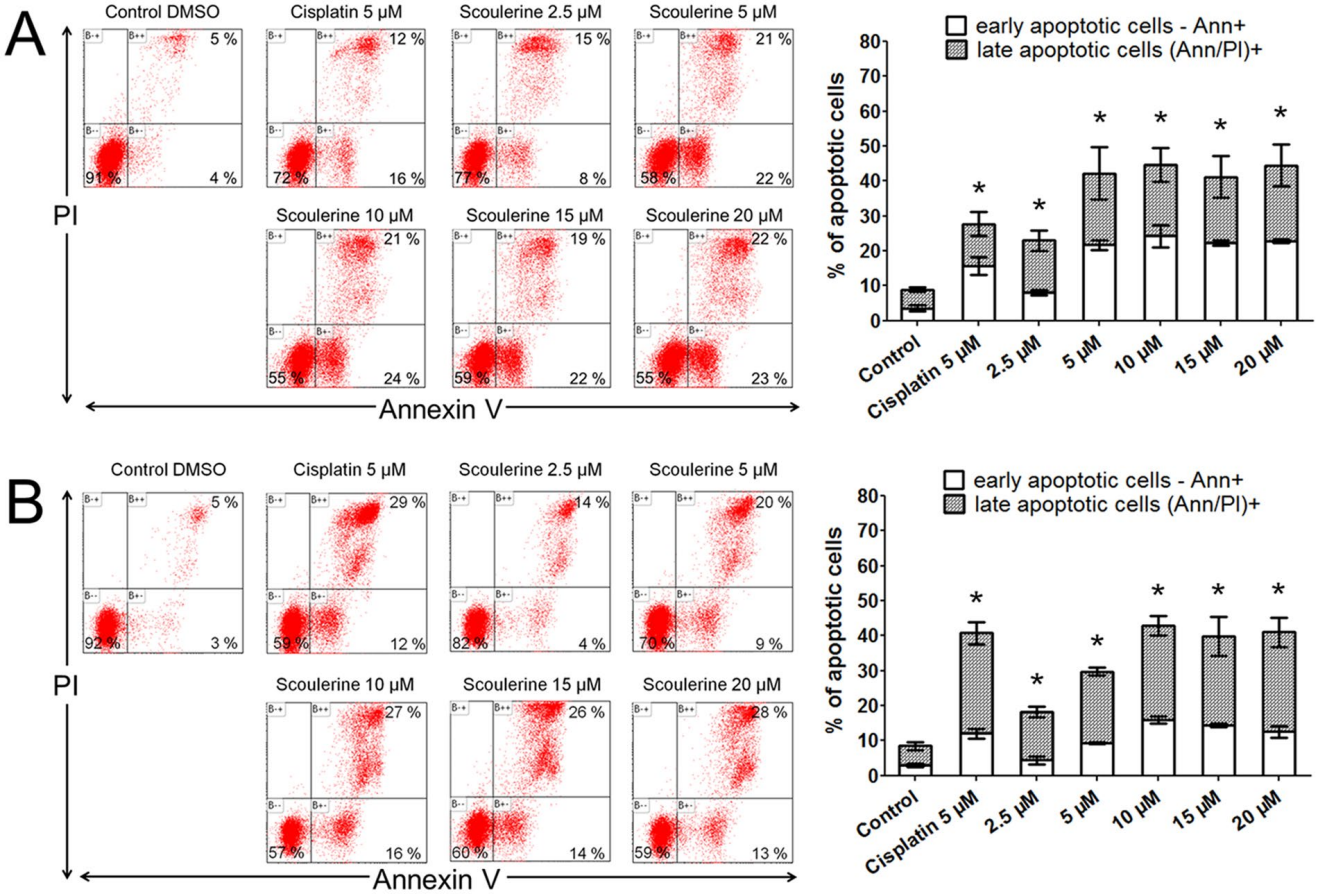
The effect of scoulerine on the induction of apoptosis in Jurkat (**A**) and MOLT-4 (**B**) leukemic cells. Apoptosis was determined by Annexin V and PI staining 24 h after treatment. Representative histograms of one of three independent measurements are shown. Cells treated with 5 µM cisplatin were used as positive control. The bar graph represents the percentage of early and late apoptotic cells detected by flow cytometry (mean ± SD, n = 3). *Significantly different to control for early and late apoptotic cells (P ≤ 0.05).

In what concerns the pro-apoptotic activity of scoulerine in both Jurkat and MOLT-4 leukemic cell lines, this compound showed an apoptosis-inducing effect assayed by Annexin V and PI staining 24 h following the treatment. Soon after initiating apoptosis, the membrane phosphatidylserine was translocated from the inner layer of the plasma membrane to the outer leaflet. Quantification of Annexin V binding to externalized phosphatidylserine allowed us to clearly identify apoptotic cells between cell populations. Twenty-four hours after the application of 0, 2.5, 5, 10, 15 and 20 µM of scoulerine, the early apoptotic rates were 4%, 8%, 22%, 24%, 22% and 23% for Jurkat cells, and 3%, 4%, 9%, 16%, 14% and 13% for MOLT-4 cells, respectively. The late apoptotic (cells that have lost their membrane integrity also show PI staining) rates for Jurkat leukemic cells were 5%, 15%, 21%, 21%, 19% and 22%, respectively, and 5%, 14%, 20%, 27%, 26% and 28% for MOLT-4, respectively (Fig. 4). In follow-up experiments, the DNA fragmentation, which is a hallmark of apoptosis due to the activation of endonucleases, was quantified by TUNEL assay. In contrast to 0.1% DMSO treated controls, Jurkat and MOLT-4 leukemic cells treated for 24 h with scoulerine showed a significant (P ≤ 0.05) dose-dependent increase in the percentage of TUNEL positive cells (Supplementary Fig. 3).

### Scoulerine activates caspase-3/7, -8 and -9 in a dose-dependent manner

**Figure 5:**
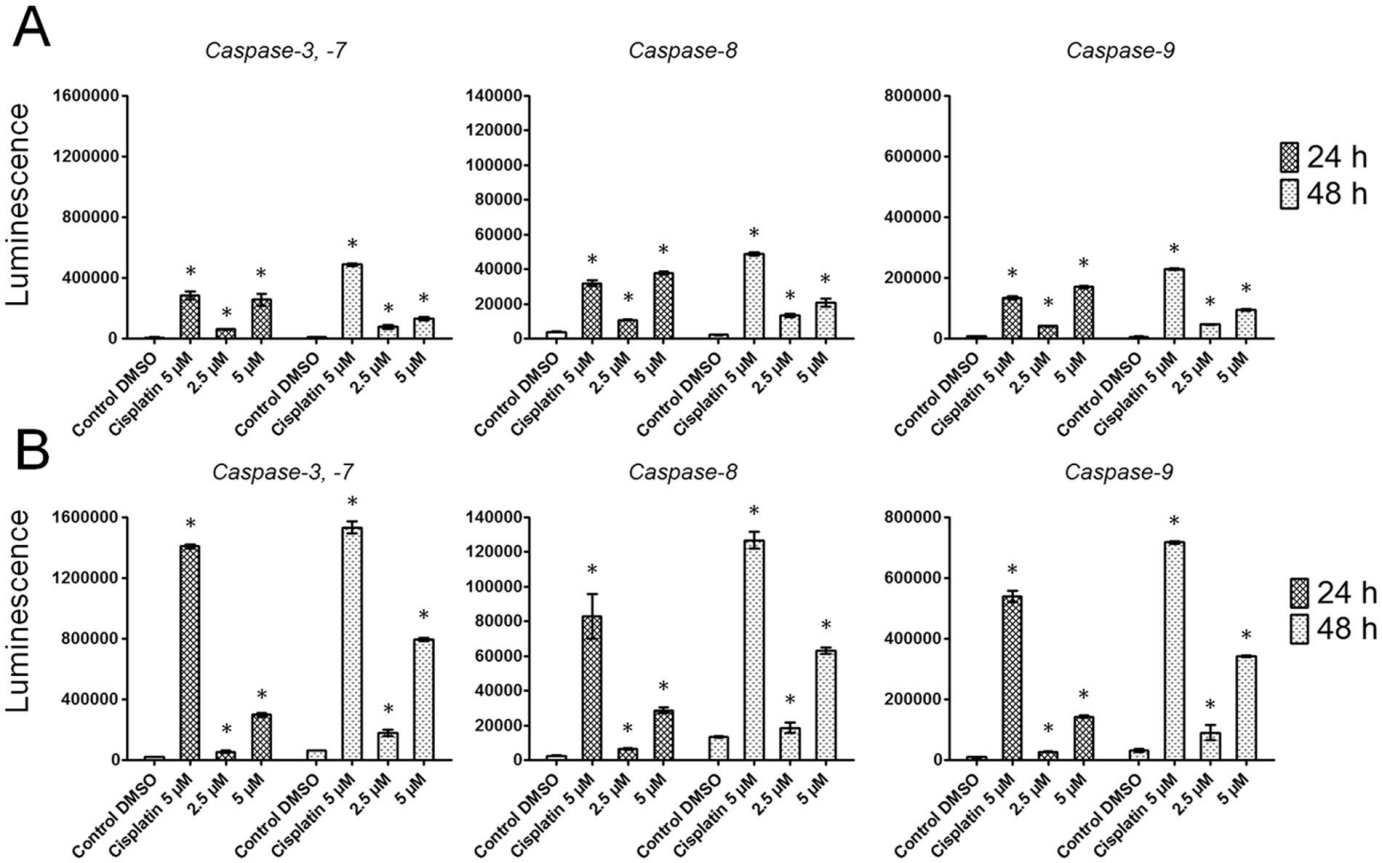
The effect of scoulerine on the activity of caspases -3/7, caspase -8 and caspase -9 was determined in Jurkat (**A**) and MOLT-4 (**B**) cells. Subsequent to treatment with scoulerine at 2.5 and 5 µM for the indicated times of 24 and 48 h, the cells were analyzed for changes in the activity of caspases-3, -8 and -9 using a luminescent assay. Cells treated with 5 µM cisplatin were used as positive control. *Significantly different to control (P ≤ 0.05).

In order to subsequently confirm apoptosis activation at a molecular level, we evaluated the activation of caspases-3/7, -8 and -9. Irrespective of which pathway of apoptosis is induced, caspases-3/7 are activated downstream of initiator caspases. Caspase-9 is activated upstream of caspases-3/7 and downstream of mitochondrial membrane permeabilization, so it indicates that the intrinsic pathway of apoptosis is being activated, while caspase-8 becomes induced after death-receptor stimulation, which is typical for extrinsically mediated apoptosis. In this case, exposure of Jurkat cells for 24 h to 2.5 and 5 µM of scoulerine resulted in considerable (P ≤ 0.05) activation of caspases-3/7 and -8, and a bit less pronounced (P ≤ 0.05) activation of caspase-9. At the 48 h interval, the activity of caspases-3/7, -8 and -9 was still statistically significant, but began to decline. On the contrary, where MOLT-4 leukemic cells were concerned, the activity of caspases-3/7, -8 and -9 was even more pronounced 48 h following the application of 2.5 and 5 µM of scoulerine, compared to the same after 24 h, with an overall higher level of activity of caspases-3/7, -8 and -9 (Fig. 5).

### Scoulerine acts to inhibit proliferation through inducing G2 or M cell cycle arrest

**Figure 6:**
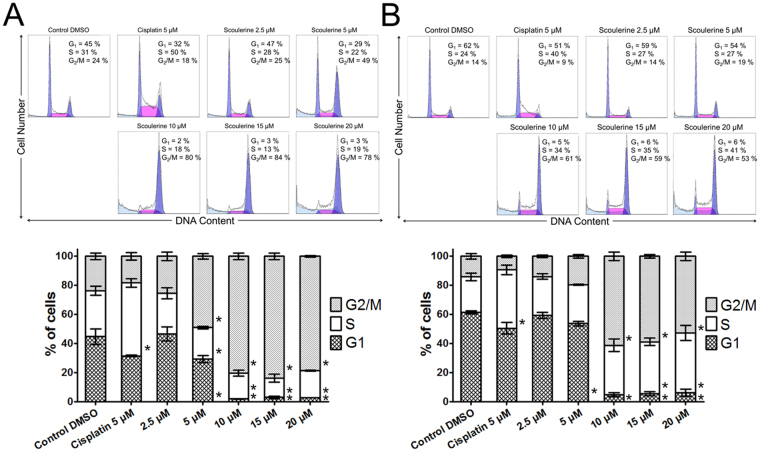
Analysis of the cell cycle of Jurkat (**A**) and MOLT-4 (**B**) leukemic cells 16 h after the application of scoulerine. Cells treated with 5 µM cisplatin were used as a reference compound. The pooled results of 1 of 3 independent experiments are shown. The bar graph represents the percentage of cells in the G1, S, and G2/M phase. Data are presented as mean values ± SD, n = 3. *Significantly different to control (P ≤ 0.05).

To further elucidate the mechanisms by which scoulerine exerts its growth-inhibitory activity, we investigated the effect of scoulerine on cell cycle progression in Jurkat and MOLT-4 leukemic cells 16 h after treatment. In the presence of 5 µM of scoulerine, the percentage of Jurkat cells in the G1phase and the S phase decreased from 45% and 31% (at mock-treated control) to 29% and 22%, respectively. Conversely, the proportion of cells in the G2/M phase increased from 24% (mock-treated control) to 49%. The rise in the percentage of Jurkat cells in the G2/M phase was even more eminent in the presence of 10, 15 and 20 µM of scoulerine. A similar trend was observed in MOLT-4 cells treated with the same amounts of scoulerine (Fig. 6). All in all, this result indicates scoulerine-induced cell cycle arrest at the G2/M transition, which was determined in later experiments by phosphorylated histone H3 at Ser10 (H3 pSer10) quantification.

**Figure 7:**
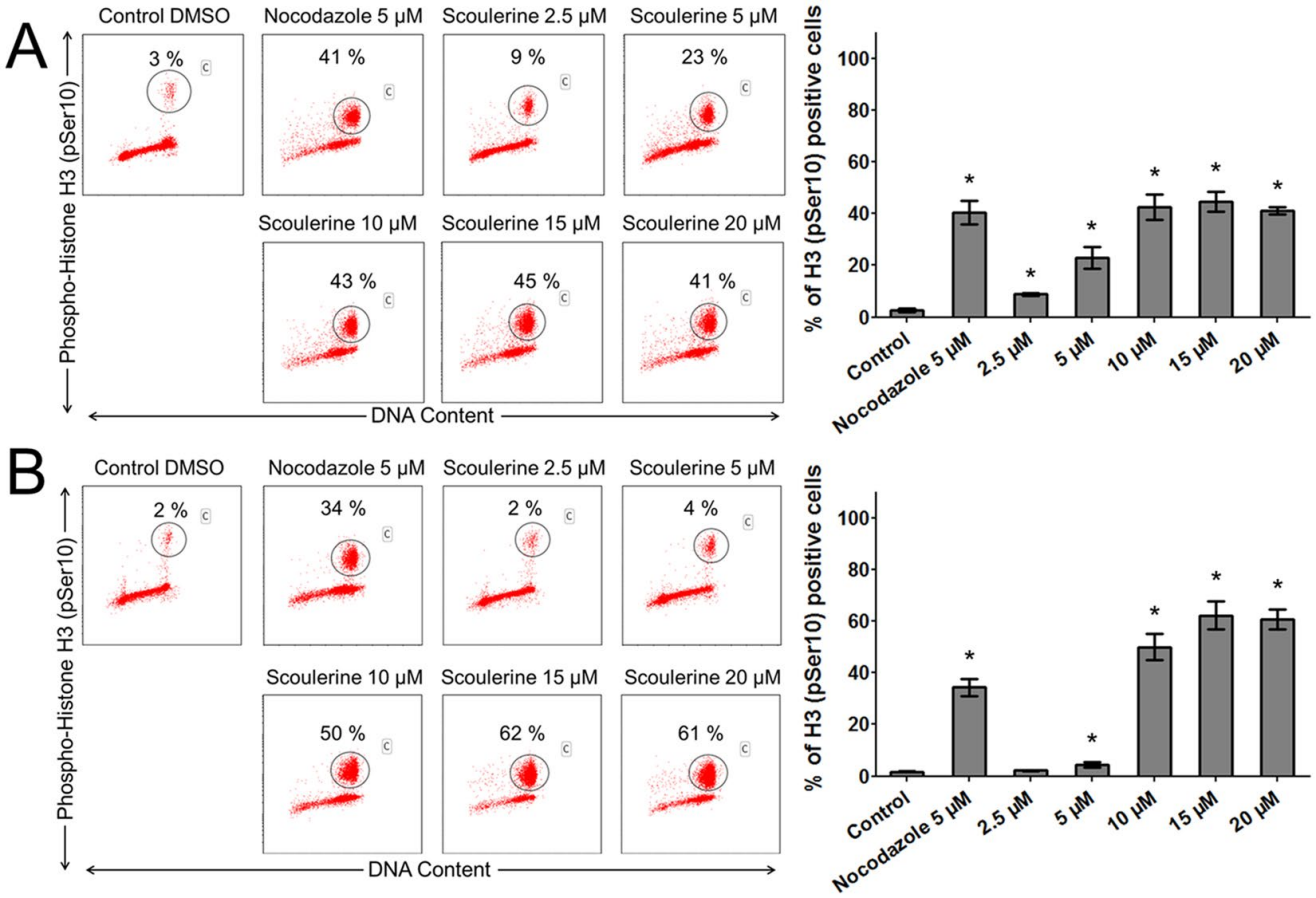
The effect of scoulerine treatment on mitotic arrest. The Jurkat (**A**) and MOLT-4 (**B**) cells were exposed to scoulerine and 5 μM of nocodazole (an antineoplastic agent that disrupts microtubule function by binding to tubulin used as a reference compound). After 24 h, pSer10 histone H3-positive population (percentages are shown) was quantified by flow cytometry. The figure shows representative flow cytometry histograms depicting Ser10-phosphorylated histone H3 positive cells (gate) in cell cultures. Bars indicate mean ± SD, n = 3. *Significantly different to corresponding control (P ≤ 0.05).

Analysis of the percentage of cells halting proliferation in mitosis (M phase) measured as positive for H3 pSer10 staining using flow cytometry revealed that scoulerine-treated Jurkat (2.5–20 µM) and MOLT-4 (5–20 µM) cells for 24 h were significantly (P ≤ 0.05) arrested in mitosis. Together, these results indicate that scoulerine is able to induce cell cycle arrest in the G2 or M phase depending on the dose and the duration of the treatment (Fig. 7).

### Scoulerine disrupts microtubule structure of A549 lung carcinoma cells

**Figure 8:**
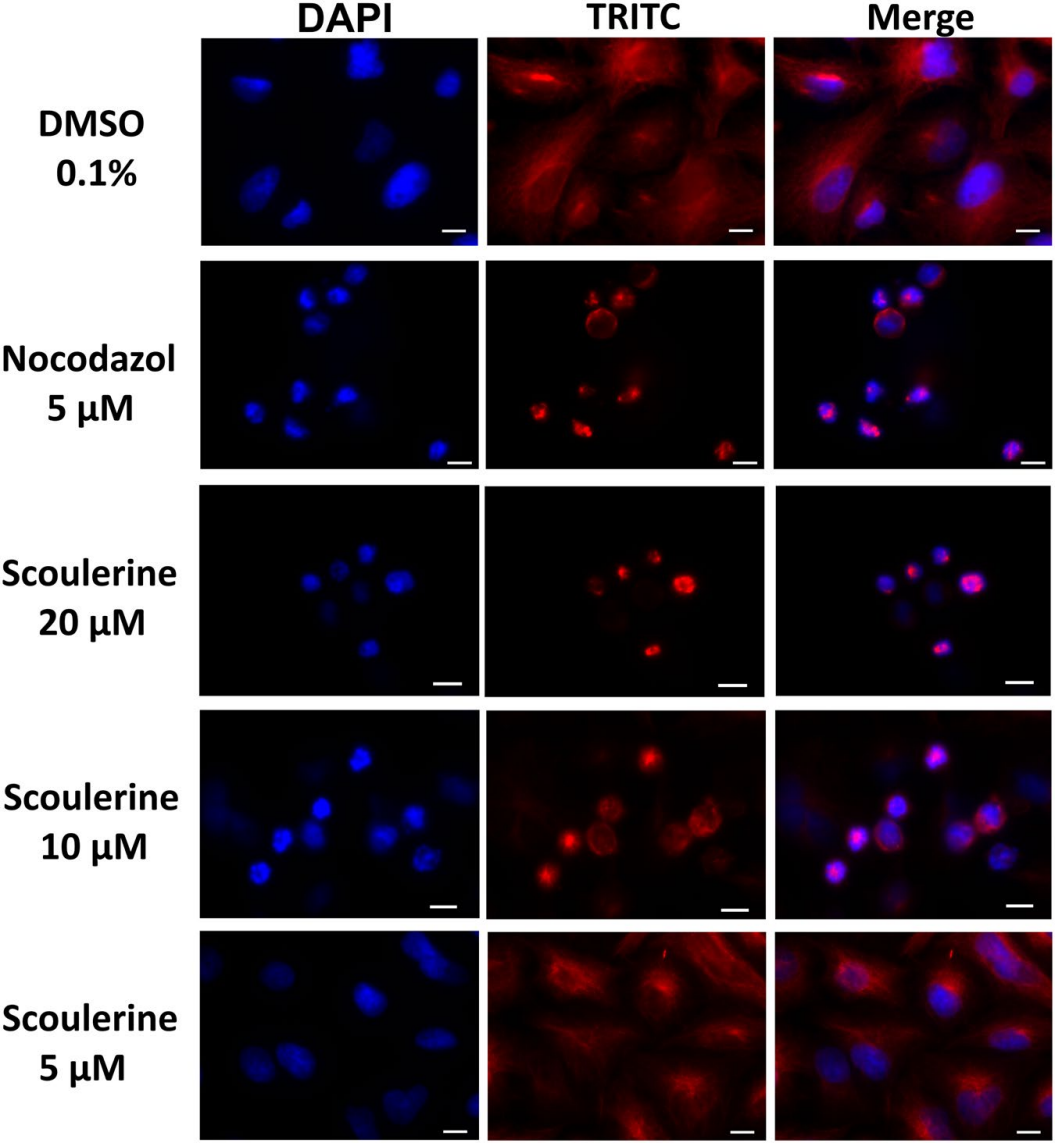
Microscopic images of A549 cells stained with an anti-β-tubulin antibody (red) and counterstained with DAPI (blue). The cells were treated for 24 h with scoulerine or a solvent (0.1% DMSO) as vehicle control. Nocodazole, an antineoplastic agent that disrupts microtubule function by binding to tubulin was used as a reference compound in this assay. Scale bar: 10 µm. Experiments were performed in triplicate using epi-fluorescence microscopy. Photographs from representative chambers are shown. Compared with controls, thicker and denser microtubule bundles were evident in scoulerine-treated cells.

Since we observed significant G2 or M phase arrest and H3 pSer10 staining during flow cytometry cell cycle analysis, we explored whether scoulerine affects the microtubule structures of cells. To visualize this, lung carcinoma A549 cells were chosen as a model for indirect immunofluorescence with a monoclonal anti-β-tubulin antibody due to a lower nuclear-cytoplasmic ratio compared to leukemic cells. Additionally, A549 cells were sensitive to scoulerine and displayed strongly diminished proliferation during xCELLigence analysis at concentrations exceeding 10 µM. Epi-fluorescence microscopy performed with fixed A549 cells labeled with a monoclonal anti-β-tubulin antibody and DAPI counter stain revealed that the intact microtubules extended continuously through the cytoplasm in control cells. In contrast, scoulerine applied for 24 h at 10 and 20 µM obviously disrupted microtubule structure with a dense aggregation of microtubules around cell nuclei, being less dense at the cell periphery. Besides, A549 cells treated with scoulerine at 5 µM resisted the treatment with almost intact organized tubulin networks as seen in 0.1% DMSO exposed control cells. Nocodazole, the microtubule depolymerizer, was used as a positive control for the study. As is apparent from the microscopic images presented in Fig. 8, treatment with nocodazole at 5 µM resulted in the observation of expected disorganized and disassembled microtubule structures as compared to 0.1% DMSO control cells.

### Scoulerine does not induce DNA strand breaks

**Figure 9:**
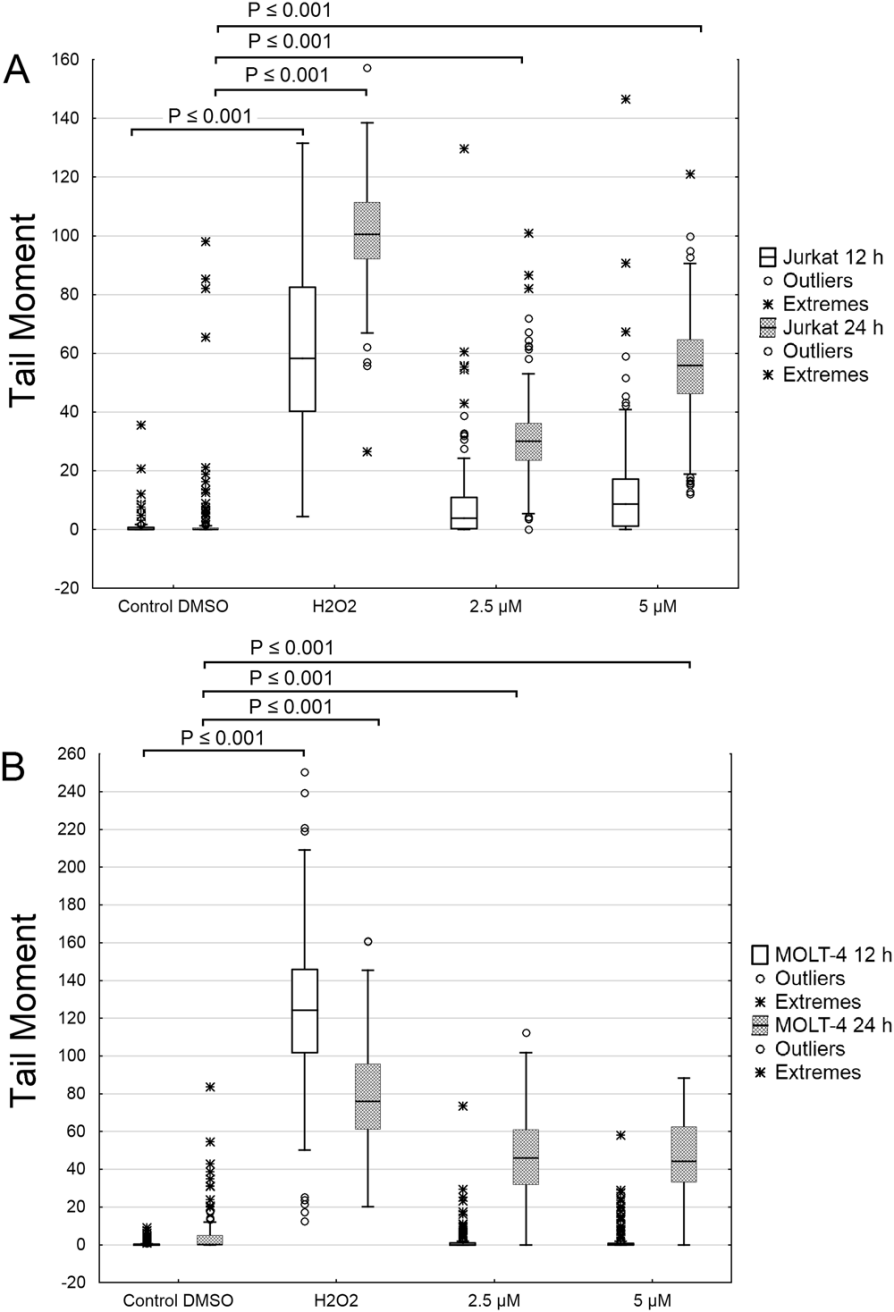
The effect of scoulerine on DNA damage. The alkaline comet assay was used for the detection of single-strand breaks of DNA in Jurkat (**A**) and MOLT-4 (**B**) cells at 12 h and 24 h following treatment with scoulerine at 2.5 and 5 µM. The 3% hydrogen peroxide was used as a positive control to verify the comet assay. The dark central bar in each box shows the median. The bottom and top of the box indicate the lower (25%) and upper (75%) quartiles, respectively. Whiskers represent the values within 1.5 interquartile ranges. Values outside this range (outliers) are plotted as solid circles. Extreme values are depicted as asterisks.

To assess DNA damage, the alkaline comet assay was performed. The treatment of Jurkat and MOLT-4 cells with scoulerine at 2.5 and 5 µM for 24 h, triggered apoptotic (secondary) DNA fragmentation, which was estimated by the formation of comet-like tails after single-cell gel electrophoresis. Briefly, in a 24 h interval, there was a significant increase in the tail moment after scoulerine application in both Jurkat and MOLT-4 leukemic cells. However, when we performed the comet assay at an earlier 12 h interval of treatment, no considerable direct DNA damage was found. This suggests that DNA damage is predominantly a consequence of internucleosomal cleavage associated with apoptotic cell death in response to scoulerine treatment (Fig. 9).

### Scoulerine alters the levels of cell cycle checkpoint and apoptosis-related proteins

**Figure 10:**
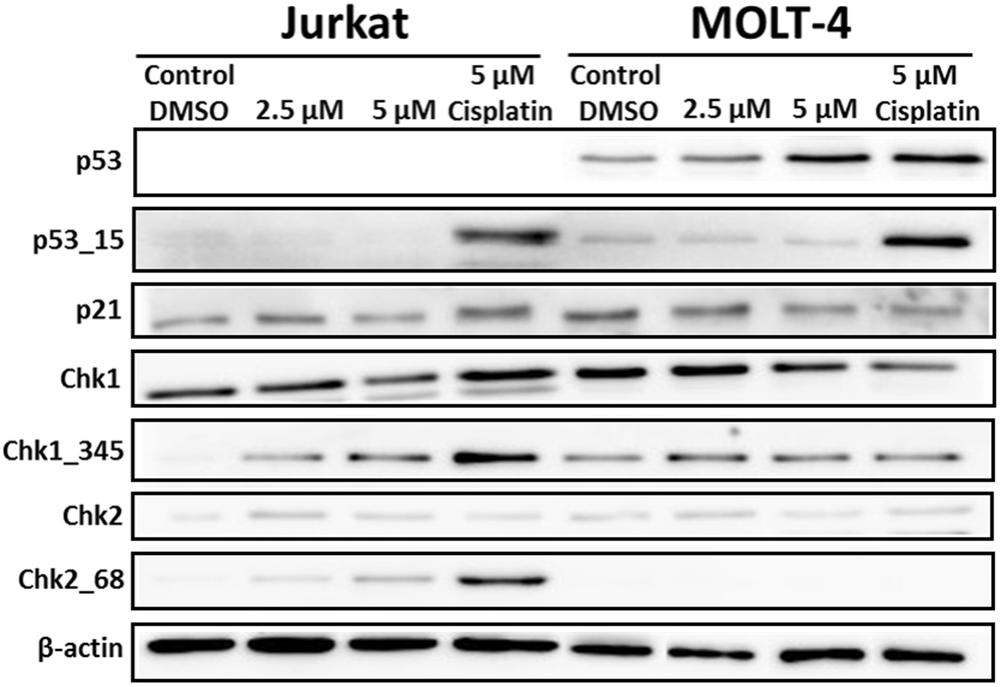
Western blot analysis of proteins that regulate cell cycle progression or cell death in Jurkat and MOLT-4 cells after scoulerine treatment for 24 h. Control cells were mock treated with 0.1% DMSO (DMSO). Cells treated with cisplatin at 5 µM were used for positive control in Western blot analysis. These experiments were performed at least three times with similar results and a cropped blot is shown from one representative experiment.

In order to study the molecular mechanism by which scoulerine induces cell cycle arrest and apoptosis, we investigated the expression levels and the activation of several cell cycle regulatory and proapoptotic proteins. To perform this, Jurkat and MOLT-4 cells were treated with scoulerine at 2.5 and 5 µM for 24 h and whole cell lysates were prepared and used for Western blotting. Western blot analysis showed an upregulation of p53 protein in p53 wild-type MOLT-4 cells in the presence of 5 µM scoulerine. In Jurkat cells, checkpoint kinase 1 (Chk1) was activated through phosphorylation at Ser345 and checkpoint kinase 2 (Chk2) was activated through phosphorylation at Thr68 after scoulerine treatment. However, in both determined cell lines the expression levels of p21, which is also involved in the regulation of cell cycle progression, remained the same. Similarly, the level of phosphorylated p53 at Ser15, which increased after cisplatin exposure (positive control), remained unchanged after scoulerine exposure (Fig. 10).

## Discussion

In our previous study we reported that scoulerine has a potent antiproliferative activity against Caco-2 and Hep-G2 cancer cells^11^. Another recent work in this direction showed that scoulerine isolated from the stems of *Xylopia laevigata* was cytotoxic toward the tumor cell lines B16-F10, HepG2, K562 and HL-60^12^. Encouraging results prompted us to investigate whether scoulerine can eliminate cancer cells via apoptosis and if the scoulerine-induced antiproliferative effect blocks cell cycle progression. Thus, in the work herein, we have investigated proliferation, cell cycle distribution, cell death, apoptosis induction, DNA damage, microtubule structure and the upregulation of selected DNA-damage response proteins following scoulerine treatment. We show that scoulerine had cytostatic activity in all of the leukemic and tumor lines investigated in a dose-dependent manner. Controversially, our results are in contrast with that reported by Khamis and colleagues. They determined only moderate cytotoxic activity of discretamine (scoulerine) with IC_50_ over 3000 µM using four human breast cancer (MCF-7, MCF-7ADR, MDA-MB435 and MT-1) cell lines and MTT assay^13^. Here, however, scoulerine inhibited the proliferation of MCF-7 cells at 10 µM, as measured by means of the xCELLigence system in view of the cell-growth inhibition profile under real-time. To better understand antiproliferative potential of this naturally occurring alkaloid, derivatives of scoulerine were synthesized and assayed at 10 µM with respect to its activity on cell growth. The comparison of the semisynthetic derivatives (**2**), (**3**) and (**4**) with scoulerine indicated that esterification generally resulted in a reduction in the antiproliferative activity. Moreover, it seems that the potencies of the (**2**), (**3**) and (**4**) esters decreased with an increasing length of the carbon chain. Next, we observed that scoulerine treatment significantly downregulated the viability of Jurkat and MOLT-4 cells within 48 h from 5 μM and higher as assessed by using a Trypan blue exclusion test. Apoptosis induction associated with the translocation of phosphatidylserine to the outside of the cell and loss of plasma membrane integrity was quantified by flow cytometry. Jurkat and MOLT-4 cell cultures treated with scoulerine showed a presence of both early and late apoptotic cells, which increased in a dose-dependent manner. Apoptosis induction was further supported by measuring DNA fragmentation using TUNEL assay, by quantifying caspase activity and by detecting p53 expression in MOLT-4 cells. Collectively, scoulerine showed caspases-3/7, -8 and -9 activation, which suggests the involvement of both an extrinsic and intrinsic pathway of programmed cell death. Further analysis by flow cytometry revealed that scoulerine treatment led to G2 phase arrest in Jurkat and MOLT-4 leukemic cells followed by an increase in the percentage of cell population in mitosis as judged by PI and phospho-histone H3 double staining. During mitotic division, the tubulin-microtubule protein system underwent assembly, which, collectively, is an essential component for chromosome segregation. Where antimitotic compounds are concerned, this protein system is a suitable target. It is these antimitotic compounds that interfere with the microtubules function by inhibiting cell proliferation in mitosis^14^. Since uncontrolled and rapid cell division is a hallmark of cancer tumor growth, microtubule-interfering agents display a remarkable efficacy in rapidly proliferating cancer cells^15^. Therefore, we sought to elucidate, using immunofluorescence imaging, whether treatment with scoulerine affects the cytoskeletal microtubule architecture of cells. The epi-fluorescence images obtained, where A549 cells were exposed to 10 and 20 μM of scoulerine for 24 h and stained with an anti-β-tubulin antibody, revealed a disruption of the tubulin structure. These results support the notion that scoulerine is a mitotic poison, resulting in G2 or M arrest. Unlike drugs grouped as genotoxic anticancer agents, our results indicate that scoulerine does not interfere with DNA, thereby activating cancer cell death independently of exogenous-induced DNA damage. However, these findings oppose what has been reported by Cheng, who proposed that scoulerine’s anticancer activity is associated with the formation of single-strand DNA breaks through the stabilization of the covalent topoisomerase I – DNA complex^10^. Thus, dissimilarly to the previous findings describing the DNA-damaging action of scoulerine, no significant DNA damage was found early at 12 h using the alkaline comet assay. However, as we expected, the later 24 h interval of treatment induced DNA fragmentation that resulted from an internucleosomal cleavage of DNA during scoulerine-induced apoptosis. Such single- or double-strand DNA breaks generated during apoptotic cell death were cross-verified by TUNEL at 24 h in both leukemic cell lines. Numerous reports have shown that replication stress and DNA damage activate critical cell-cycle checkpoint kinases Chk1 and Chk2 of the DNA damage response pathway^16,17^. Both Chk1 phosphorylated at Ser345 by Ataxia telangiectasia and Rad3-related (ATR) kinase and Chk2 phosphorylated at Thr68 by Ataxia telangiectasia mutated (ATM) kinase were determined after 24 h of treatment. These results imply that activated Chk1 and Chk2 are particularly important to pause cell cycle progression after scoulerine exposure.

Although scoulerine has been studied for several years, only few publications regarding its cytotoxic activity against mammalian cells exist. The most explored part is its biosynthesis in the plants of the Fumaridaceae family. The biosynthesis of scoulerine begins with the condensation of two tyrosine derivatives, dopamine and 4-hydroxyphenylacetaldehyde, yielding 1-benzylisoquinoline (*S*)-norcoclaurine as the central precursor for the biosynthesis. Internal carbon-carbon coupling of the (*S*)-norcoclaurine derivative results in the formation of branch point intermediate (*S*)-reticuline. Then stereo-specific conversion changes of the central intermediate (*S*)-reticuline to (*S*)-scoulerine and the formation of (*S*)-scoulerine is the first step in the biosynthesis of the subsequent benzo[c]phenanthridine, protopine and protoberberine alkaloids, sanguinarine, protopine and berberine, respectively^6,18^. Among these, it is important to point out that sanguinarine, protopine and berberine were reported to show various degrees of cytotoxicity and antiproliferative activity against cancer cells. Although sanguinarine did not change the proportion of mitotic cells, it inhibited tubulin polymerization leading to the disruption of the microtubule network^19^. Interestingly, protopine is able to target microtubule structures in living cells without affecting tubulin polymerization with cell cycle arrest at G2/M^20^. In the same manner, disruptions of the microtubule network were described in response to berberine treatment^21^. Even if this structurally related substance shares some mechanistic similarities, it should be noted that these compounds differ in growth inhibitory values and molecular actions, thus making them an attractive target for structure-activity relationship studies.

In summary, scoulerine was shown to be broadly cytostatic and cytotoxic in the range of micromolar concentrations. To further investigate the underlying MOA, we showed that scoulerine caused a pronounced accumulation of cells in the G2 or M phases of the cell cycle, increased phosphorylation of histone H3 at Ser10 and disruption of microtubule organization, which is consistent with the antimitotic mechanism of action. Additionally, scoulerine has shown molecular target activity that occurs during apoptosis, with a p53 protein increase, caspases-3/7, -8 and -9 activation, phosphatidylserine externalization and DNA fragmentation. Our findings suggested that scoulerine activated ATR and ATM kinase-dependent cell cycle checkpoint signaling followed by phosphorylation of Chk1 at Ser345 and Chk2 at Thr68 sites. To our best knowledge, this is the first example to show the potent antimitotic activity of scoulerine leading to microtubule disruption, cell cycle arrest and cell death via apoptosis induction in cancer cells. Altogether, our results identified isoquinoline alkaloid scoulerine as a potent microtubule targeting agent, and data on biochemical interactions playing an important role in the cytotoxic, antiproliferative and proapoptotic actions are sufficiently encouraging to warrant further research on its anticancer potential.

## Methods

### General experimental procedures

All reagents were purchased from commercial sources (Acros Organics, Morris Plains, NJ, USA; Sigma Aldrich, St. Louis, MO, USA) and used without purifications. NMR spectra were recorded for CDCl_3_ and CD_3_OD solutions at ambient temperature on a VNMR S500 NMR (Varian, Palo Alto, CA, USA) spectrometer operating at 500 MHz for ^1^H and 125 MHz for ^13^C. Chemical shifts were recorded as δ values in parts per million (ppm), and were indirectly referenced to tetramethylsilane (TMS) *via* the solvent signal (7.26 ppm for ^1^H and 77.0 ppm for ^13^C for CDCl_3_, and 3.30 ppm for ^1^H and 49.0 ppm for ^13^C for CD_3_OD). Coupling constants (*J*) are given in Hz. For unambiguous assignment of ^1^H and ^13^C signals 2D NMR spectra (COSY, gHSQC, gHMBC and NOESY) were measured using standard parameter settings and pulse programs delivered by the producer of the spectrometer. ESI-HRMS were obtained with a Waters Synapt G7-Si with a hybrid mass analyzer quadrupole-time-of-flight (Q-TOF), coupled to a Waters Acquity I-Class UHPLC system. The EI-MS were obtained on an Agilent 7890 A GC 5975 inert MSD operating in EI mode at 70 eV (Agilent Technologies, Santa Clara, CA, USA). A DB-5 column (30 m × 0.25 mm × 0.25 μm, Agilent Technologies, Santa Clara, CA, USA) was used. The temperature program was: 100–180 °C at 15 °C/min, 1 min hold at 180 °C, and 180–300 °C at 5 °C/min and 5 min hold at 300 °C; detection range *m/z* 40–600. The injector temperature was 280 °C. The flow-rate of carrier gas (helium) was 0.8 mL/min. A split ratio of 1:15 was used. TLC was carried out on Merck precoated silica gel 60 F_254_ plates. Compounds on the plate were observed under UV light (254 and 366 nm) and visualized by spraying with Dragendorff´s reagent.

### Scoulerine and its esters

Scoulerine (**1**) has been previously isolated from dry tubers of *Corydalis cava*^4^. Results of GC/MS, HPLC, ^1^H-NMR and ^13^C-NMR analysis are shown in Supplementary Figs 4, 5, 6 and 7.

### General procedure for acylation of scoulerine

The 1.5 equiv. of the corresponding anhydride was added to a solution of scoulerine (**1**) in 3 mL of dry pyridine. The mixture was stirred at room temperature until disappearance of the starting material. Then, the solvent was evaporated and the residue was purified by preparative TLC using cHx:Et_2_NH 9:1 or cHx:To:Et_2_NH 45:45:10 to afford corresponding esters **2**–**4**^22^.

### 2,9-Di-*O*-Acetylscoulerine (2)

Following the procedure described above, 17 mg (0.05 mM) of **1** was treated with 0,2 mL of acetic anhydride. After purification, 18 mg of **2** (84%) were obtained as an orange oil. ^1^H NMR (500 MHz, CDCl_3_) δ: 7.01 (1 H, d, *J* = 8.3 Hz), 6.93 (1 H, s), 6.84 (1 H, d, *J* = 8.3 Hz), 6.71 (1 H, s), 4.03 (1 H, d, *J* = 15.6 Hz), 3.83 (3 H, s), 3.82 (3 H, s), 3.59 (1 H, d, *J* = 11.5 Hz), 3.45 (1 H, d, *J* = 15.6 Hz), 3.23 (1 H, dd, overlapped, *J* = 16.1 Hz, *J* = 3.4 Hz), 3.21–3.12 (2 H, m, overlapped), 2.86 (1 H, dd, *J* = 15.6 Hz, *J* = 11.5 Hz), 2.79–2.70 (1 H, m), 2.69–2.61 (1 H, m), 2.35 (3 H, s), 2.33 (3 H, s). ^13^C NMR (125 MHz, CDCl_3_) δ: 169.2, 168.6, 149.2, 149.0, 138.0, 136.1, 133.0, 130.0, 128.1, 127.5, 126.4, 119.7, 112.3, 110.6, 58.6, 56.0, 55.9, 53.3, 51.0, 35.8, 29.4, 20.7, 20.4. [α]^24^_D_ = −198 (*c* = 0.171; MeOH); EIMS m/z (%) 411 (41), 410 (52), 352 (50), 176 (23), 150 (100). HRESIMS m/z calcd for C_23_H_26_NO_6_ (M + H^+^) 412.1760 found 412.176. Results of GC/MS, HPLC, ^1^H-NMR, ^13^C-NMR and HRMS analysis are shown in Supplementary Figs 8, 9, 10, 11 and 12.

### 2,9-Di-O-Propionylscoulerine (3)

Following the procedure described above, 26 mg (0.08 mM) of **1** was treated with 0,2 mL of propionic anhydride. After purification, 21 mg of **3** (60%) were obtained as an orange oil. ^1^H NMR (500 MHz, CDCl_3_) δ: 7.00 (1 H, d, *J* = 8.4 Hz), 6.92 (1 H, s), 6.83 (1 H, d, *J* = 8.4 Hz), 6.70 (1 H, s), 4.01 (1 H, d, *J* = 15.4 Hz), 3.81 (3 H, s), 3.80 (3 H, s), 3.58 (1 H, dd, *J* = 11.3 Hz, *J* = 3.6 Hz), 3.44 (1 H, d, *J* = 15.4 Hz), 3.23 (1 H, dd, *J* = 15.4 Hz, *J* = 3.6 Hz), 3.21–3.11 (2 H, m), 2.85 (1 H, dd, *J* = 15.4 Hz, *J* = 11.3 Hz), 2.76–2.69 (1 H, m), 2.68–2.61 (1 H, m, overlapped), 2.65 (2 H, q, overlapped, *J* = 7.7 Hz), 2.63 (2 H, q, overlapped, *J* = 7.7 Hz), 1.32 (3 H, t, overlapped, *J* = 7.7 Hz), 1.30 (3 H, t, overlapped, *J* = 7.7 Hz). ^13^C NMR (125 MHz, CDCl_3_) δ: 172.7, 172.1, 149.2, 149.0, 138.1, 136.1, 132.9, 130.0, 128.1, 127.5, 126.3, 119.7, 112.2, 110.5, 58.6, 56.0, 55.9, 53.3, 51.1, 35.8, 29.4, 27.3, 27.2, 9.3, 9.1. [α]^24^_D_ = −147 (*c* = 0.115; MeOH); EIMS m/z (%) 439 (21), 438 (46), 382 (14), 366 (66), 326 (9), 176 (25), 149 (100). HRESIMS m/z calcd for C_25_H_30_NO_6_ (M + H^+^) 440.2073 found 440.2071. Results of GC/MS, HPLC, ^1^H-NMR, ^13^C-NMR and HRMS analysis are shown in Supplementary Figs 13, 14, 15, 16 and 17.

### 2,9-Di-O-Hexanoylscoulerine (4)

Following the procedure described above, 19 mg (0,06 mM) of **1** was treated with 0,2 mL of hexanoic anhydride. After purification, 23 mg of **4** (76%) were obtained as an orange oil. ^1^H NMR (500 MHz, CDCl_3_) δ: 7.00 (1 H, d, *J* = 8.3 Hz), 6.91 (1 H, s), 6.83 (1 H, d, *J* = 8.3 Hz), 6.70 (1 H, s), 4.01 (1 H, d, *J* = 15.5 Hz), 3.81 (3 H, s), 3.80 (3 H, s), 3.59 (1 H, dd, *J* = 11.3 Hz, *J* = 2.9 Hz), 3.44 (1 H, d, *J* = 15.5 Hz), 3.23 (1 H, dd, overlapped, *J* = 15.9 Hz, *J* = 3.7 Hz), 3.21–3.11 (2 H, m, overlapped), 2.86 (1 H, dd, *J* = 15.6 Hz, *J* = 11.3 Hz), 2.77–2.69 (1 H, m), 2.68–2.64 (1 H, m, overlapped) 2.61 (2 H, t, overlapped, *J* = 7.3 Hz), 2.59 (2 H, t, overlapped, *J* = 7.3 Hz), 1.85–1.76 (4 H, m), 1.49–1.36 (8 H, m), 0.95 (6 H, dt, *J* = 7.1 Hz, *J* = 2.9 Hz). ^13^C NMR (125 MHz, CDCl_3_) δ: 172.1, 171.4, 149.2, 149.0, 138.1, 136.1, 132.8, 130.0, 128.1, 127.4, 126.3, 119.7, 112.2, 110.5, 58.6, 56.0, 55.8, 53.4, 51.1, 35.8, 34.0, 33.9, 31.3, 31.2, 29.4, 24.8, 24.7, 22.3, 13.9, 13.9. [α]^24^_D_ = −200 (*c* = 0.141; MeOH); ESIMS m/z (%) [M + H^+^] 524 (100). HRESIMS m/z calcd for C_31_H_42_NO_6_ (M + H^+^) 524.3012 found 524.3013. Results of GC/MS, HPLC, ^1^H-NMR, ^13^C-NMR and HRMS analysis are shown in Supplementary Figs 18, 19, 20 and 21.

The purity of all compounds verified by NMR was ≥97%.

### Cell cultures and culture conditions

Jurkat, Raji and A2780 cells were propagated in RPMI 1640 medium supplemented with 10% foetal bovine serum, 2 mM L-glutamine, 1 mM pyruvate, 10 mM HEPES, MEM Non-Essential Amino Acids 10 µl/ml, 50 µg/ml penicillin and 50 µg/ml streptomycin (all reagents from Life Technologies, Grand Island, NY, USA). MOLT-4 cells were cultured in RPMI 1640 medium supplemented with 20% foetal calf serum, 2 mM L-glutamine, 1 mM pyruvate, 10 mM HEPES, MEM Non-Essential Amino Acids 10 µL/mL, 50 µg/mL penicillin and 50 µg/mL streptomycin (all reagents from Life Technologies, Grand Island, NY, USA). HEL 92.1.7 cells were cultured in RPMI 1640 medium supplemented with 10% foetal calf serum, 2 mM L-glutamine, 50 µg/mL penicillin and 50 µg/mL streptomycin (all reagents from Life Technologies, Grand Island, NY, USA). HL-60 cells were cultured in RPMI 1640 medium supplemented with 20% foetal calf serum, 2 mM L-glutamine, 50 µg/ml penicillin and 50 µg/ml streptomycin (all reagents from Life Technologies, Grand Island, NY, USA). U-937 cells were cultured in RPMI 1640 medium supplemented with 10% foetal calf serum, 2 mM L-glutamine, 1 mM pyruvate, 10 mM HEPES, 50 µg/ml penicillin and 50 µg/ml streptomycin (all reagents from Life Technologies, Grand Island, NY, USA). A549 cells were cultured in Minimum Essential Medium Eagle with L-glutamine and sodium bicarbonate (Sigma-Aldrich, St. Louis, MO, USA) in the presence of 10% foetal calf serum, 1 mM pyruvate, 10 mM HEPES, 50 µg/ml penicillin and 50 µg/ml streptomycin (all supplements from Life Technologies, Grand Island, NY, USA). SK-BR-3 cells were cultured in McCoy’s 5 A medium (Life Technologies, Grand Island, NY, USA) supplemented with 10% foetal bovine serum and 50 µg/ml penicillin/streptomycin (all reagents and supplements from Life Technologies, Grand Island, NY, USA). MCF-7 cells were maintained in Minimum Essential Medium Eagle with L-glutamine and sodium bicarbonate (Sigma-Aldrich, St. Louis, MO, USA) supplemented with 10% foetal calf serum, 1 µg/ml insulin, 50 µg/ml penicillin and 50 µg/ml streptomycin (all reagents from Life Technologies, Grand Island, NY, USA). Jurkat, MOLT-4, Raji, HL-60, U-937, HEL 92.1.7, A549, A2780, SK-BR-3 and MCF-7 cells were purchased from the European Collection of Cell Cultures (ECACC, Salisbury, UK). The cell cultures were maintained under standard cell culture conditions at 37 °C in a humidified incubator in an atmosphere of 5% CO_2_–95% air. Cells were passaged every 2–3 days to obtain exponential growth. The cells were maintained in culture no more than 20 passage.

### Cell treatment

Scoulerine; (−)-scoulerine: fresh stock solutions of scoulerine in concentrations of 50 mM were dissolved in dimethyl sulfoxide - DMSO (Sigma-Aldrich, St. Louis, MO, USA). Stock solutions were freshly prepared before use in the experiments. Scoulerine was isolated from tubers of *Corydalis cava* (L.) Schweigg. et Koerte (Fumariaceae). The detailed isolation and identification (1D-, 2D-NMR and MS experiments) of alkaloid is described in^4^. Esters of (-)-scoulerine were dissolved in dimethyl sulfoxide - DMSO (Sigma-Aldrich, St. Louis, MO, USA) at 50 mM. For the experiments, the stock solutions were diluted with the complete culture medium to create final concentrations of 1–50 μM, making sure the concentration of DMSO was <0.1% to avoid the toxic effects on the cells. Cisplatin, doxorubicin and nocodazole were purchased from Sigma-Aldrich (Sigma-Aldrich, St. Louis, MO, USA). Control cells were sham-treated with a DMSO vehicle only (0.1%; control). Cells treated with 5% DMSO, cisplatin at 5 µM, doxorubicin at 1 µM or nocodazole at 5 µM were used as positive control.

### Cytotoxicity screening using XTT assay

In order to determine cell viability of cells treated with scoulerine, alkaloid esters (a single-dose of 10 µM) or scoulerine in a broad concentration range we used a standard colorimetric method measuring a tetrazolium salt reduction via mitochondrial dehydrogenase activity. The cells were seeded at previously established optimal density in a 96-well plate. After 48 hours incubation cell viability was determined using Cell Proliferation Kit II (XTT, Roche, Germany) according to manufacturer’s instructions. XTT-assay was conducted using 200 μl of volume and 100 μl of XTT-labeling mixture. Absorbance was then measured at 480 nm using a 96-multiwell microplate reader Tecan Infinite M200 (Tecan Group Ltd., Männedorf, Switzerland). Viability was calculated as described in the paper by Havelek and colleagues using the following formula: (%) viability = (A480sample − A480blank)/(A480control − A480blank) × 100, where A480 is the absorbance of utilized XTT formazan measured at 480 nm^23^. Data were analysed with GraphPad Prism 5 biostatistics (GraphPad Software, La Jolla, CA, USA) statistical software. Each value is the mean of at least three independent replicates of each condition.

### Screening for antiproliferative activity using xCELLigence system

The xCELLigence system (Roche, Basel, Switzerland and ACEA Biosciences, San Diego, CA, USA) was used to monitor cell adhesion, proliferation and cytotoxicity. The xCELLigence system was connected and tested by Resistor Plate before the RTCA Single Plate station was placed inside the incubator at 37 °C and 5% CO_2_. First, the optimal seeding concentration for experiments was optimized for each cell line. After seeding, the respective number of cells in 190 µL medium per each well of the E-plate 96, the proliferation, attachment and spreading of the cells were monitored every 30 minutes by the xCELLigence system. Approximately 24 h after seeding, when the cells were in the log growth phase, the cells were exposed in triplicates to 10 µL sterile deionized water containing scoulerine to obtain final concentrations 1–50 μM. Controls received sterile deionized water + DMSO with a final concentration of 0.1%. Cells treated with 5% DMSO were used as positive control. Growth curves were normalized to the time point of treatment. Evaluations were performed using xCELLigence 1.2.1 software (Roche, Basel, Switzerland and ACEA Biosciences, San Diego, CA, USA).

### Proliferation and viability

Cell proliferation and viability of Jurkat and MOLT-4 cells were determined 24 and 48 h after treatment with 2.5, 5, 10, 15 and 20 μM of scoulerine. Cells treated with 5 µM cisplatin were used as positive control. Cell membrane integrity was determined using the Trypan blue exclusion technique – mixing 10 μl of 0.4% Trypan blue and 10 μl of cell suspension. Cell counts were carried out using a Bürker chamber and light microscope Nikon Eclipse E200 (Nikon, Tokyo, Japan).

### Analysis of apoptosis

Apoptosis was determined by flow cytometry using an Alexa Fluor® 488 Annexin V/Dead Cell Apoptosis kit (Life Technologies, Grand Island, NY, USA) according to the manufacturer’s instructions. The Alexa Fluor® 488 Annexin V/Dead Cell Apoptosis kit employs the property of Alexa Fluor® 488 conjugated to Annexin V to bind to phosphatidylserine in the presence of Ca^2+^, and the property of propidium iodide (PI) to enter cells with damaged cell membranes and to bind to DNA^24^. Measurement was performed immediately using a CyAn (Beckman Coulter, Miami, FL, USA) flow cytometer. Listmode data were analysed using Kaluza Analysis 1.3 software (Beckman Coulter, Miami, FL, USA).

### TUNEL assay

To determine apoptosis by TUNEL assay, 1 × 10^6^ cells were collected and evaluated using a DNA breaks labelling detection kit, APO-DIRECT (Merck Millipore, Billerica, MA, USA) in accordance with the manufacturer’s instructions^25^. Cells were washed twice with PBS at 4 °C. Then, 1 mL of fixation solution was added to the cell suspension (1% formaldehyde) for 30 min on ice. Cells were centrifuged (5 min, 300 g.) and washed twice with 5 mL PBS. Cells were permeabilized in 70% ethanol for at least 24 h at −20 °C. Cells were washed twice with wash buffer and incubated in a TUNEL reaction mixture (dUTP-FITC; TdT enzyme, TdT reaction buffer, distilled H_2_O) for 60 min at 37 °C. Cells were washed again twice with rinse buffer and resuspended in 0.5 mL propidium iodide/RNase A solution. Samples were analysed in a CyAn ADP (Beckman Coulter, Miami, FL, USA) flow cytometer, measuring green (dUTP-FITC incorporated in fragmented DNA) and red (PI binding to DNA) fluorescence of nuclei of individual cells. Listmode data were analysed using Kaluza Analysis 1.3 software (Beckman Coulter, Miami, FL, USA).

### Cell cycle distribution and internucleosomal DNA fragmentation analysis

Where cell cycle distribution analysis is concerned, the cells were washed with ice cold PBS and fixed with 70% ethanol. In order to detect low molecular-weight fragments of DNA, the cells were incubated for 5 minutes at room temperature in a buffer (192 ml 0.2 M Na_2_HPO_4_ + 8 ml of 0.1 M citric acid, pH 7.8) and then labelled with propidium iodide in Vindelov’s solution for 1 h at 37 °C^24^. DNA content was determined using the flow cytometer CyAn (Beckman Coulter, Miami, FL, USA) with an excitation wave length of 488 nm. The data were analysed using Multicycle AV software (Phoenix Flow Systems, San Diego, CA, USA).

### Activity of caspases

The induction of programmed cell death was determined by monitoring the activities of caspases 3/7, caspase 8 and caspase 9 by Caspase-Glo Assays (Promega, Madison, WI, USA) 24 and 48 h after treatment with 2.5 and 5 μM of scoulerine. Cells treated with 5 µM cisplatin were used as positive control. The assay provides a proluminogenic substrate in an optimized buffer system. The addition of a Caspase-Glo Reagent results in cell lysis, followed by caspase cleavage of the substrate and the generation of a luminescent signal. A total of 1 × 10^4^ cells were seeded per well using a 96-well-plate format (Sigma-Aldrich, St. Louis, MO, USA). After treatment, the Caspase-Glo Assay Reagent was added to each well (50 μl/well) and incubated for 30 minutes before luminescence was measured using a Tecan Infinite M200 spectrometer (Tecan Group, Männedorf, Switzerland).

### Enumeration of mitotic cells, including measurement of histone H3 phosphorylated at Ser10 and DNA content

Histone H3 is phosphorylated at Ser10 when cells enter the mitotic phase and remains unphosphorylated in the other phases of the cell cycle. To determine the number of mitotic cells, cells treated with scoulerine for 24 h were fixed and permeabilized by adding ice-cold 100% methanol. Following overnight incubation, the cells were washed with a PBS-0.5% BSA solution and subsequently stained with the Alexa Fluor 488-conjugated anti-pSer10-histone H3 (Cell Signaling Technology, Beverly, MA, USA) antibody diluted with a PBS-0.5% BSA solution in accordance with the manufacturer’s instructions. Cells were incubated with agitation for 60 minutes at room temperature in the dark. Finally, the cells were washed twice with the PBS-0.5% BSA solution and resuspended in a PI staining solution (BD Biosciences, San Jose, CA, USA) at a final volume of 500 µL. All samples were immediately analyzed by a CyAn flow cytometer and the data were plotted using Kaluza Analysis 1.3 software (both from Beckman Coulter, Miami, FL, USA).

### Epi-fluorescence microscopy

For each condition, 250 000 cells were seeded in 2-well chamber slides SPL (SPL Life Sciences, Korea). After seeding (usually 24 h later), spent medium was replaced with fresh medium and the cells were treated with scoulerine at 5, 10 and 20 µM. Cells treated with 5 µM nocodazole were used as positive control. Following 24-h treatment the cells were fixed with 4% freshly prepared paraformaldehyde for 10 minutes at room temperature, washed with PBS, permeabilized in 0.2% Triton X-100/PBS for 15 minutes at room temperature and washed with PBS (all reagents from Sigma–Aldrich, St. Louis, MO, USA). Before incubation with the primary antibody (overnight at 4 °C), the cells were incubated with 7% heat inactivated foetal calf serum +2% bovine serum albumin in PBS for 30 minutes at room temperature. Mouse monoclonal anti-β-tubulin (Life Technologies, Grand Island, NY, USA) was used for β-tubulin detection. For the secondary antibody, the affinity pure donkey anti-mouse-TRITC-conjugated antibody was purchased from the Jackson ImmunoResearch Laboratories (West Grove, PA, USA). The secondary antibody was applied to each slide (after their pre-incubation with 5.5% donkey serum in PBS for 30 minutes at room temperature), incubated for 1 h in the dark and washed (3 × 5 min) with PBS. The nuclei were counterstained with 100 µl of 4′,6-diamidino-2-phenylindole (DAPI) at 1 µg/ml for 30 min. After the last two washes with PBS, the slides were mounted with an antifading ProLong® Gold mounting medium (Life Technologies, Grand Island, NY, USA). Images of all of the examined slides were obtained by a Nikon epi-fluorescence microscope system Eclipse 80i; the exposure time and dynamic range of the camera in all of the channels were adjusted to the same values for all of the slides to portray quantitatively comparable images. Images were further processed and merged using NIS-Elements Advanced Research 4.13 (instrument and software from Nikon, Tokyo, Japan).

### Comet assay

DNA damage was measured using alkaline version of comet assay. Briefly, cells embedded in 1% agarose (Sigma–Aldrich, St. Louis, MO, USA) on microscope slides were lysed in 10 mM Tris-buffered 2.5 M NaCl (pH 10.0; Penta, Prague, Czech Republic) containing 1% Triton × 100 (Merck Millipore, Billerica, MA, USA) and 100 mM EDTA (Penta, Prague, Czech Republic) for 1 hour at 4 °C. In alkaline conditions (NaOH, EDTA), the electrophoresis was carried out at 40 V, 300 mA, for 30 minutes at 4 °C after 40 minutes of unwinding. DNA damage was analyzed by the comet module of Lucia 6.20 image analysis (Laboratory Imaging, Prague, Czech Republic) after the cells were stained with ethidium bromide (Sigma–Aldrich, St. Louis, MO, USA). The percentage of DNA in the comet tail was measured. At least fifty cells per slide were analyzed.

### Western blot analysis

Whole-cell lysates (Cell Lysis Buffer, Cell Signaling Technology, Danvers, MA, USA) were prepared 24 h following treatment of Jurkat and MOLT-4 cells with scoulerine. Cells treated with 0.1% DMSO were used as negative control and cells treated with 5 µM of cisplatin were used as positive control. Quantification of the protein content was performed using the BCA assay (Sigma-Aldrich, St. Louis, MO, USA). The lysates (20 μg purified protein) were loaded into each lane of a polyacrylamide gel. After electrophoretic separation, the proteins were transferred to a PVDF membrane (Bio-Rad, Hercules, CA, USA). Non-specific binding of the membranes was blocked for 1 h in a Tris-buffered saline containing 0.05% Tween 20 (TBS) and 5% non-fat dry milk. The membranes were washed in TBS. Incubation with a primary antibody against specific antigens (p53, p53_serine 15 – Exbio, Prague, Czech Republic; β-actin – Sigma-Aldrich, St. Louis, MO, USA; Chk1, Chk1_serine 345, Chk2, Chk2_ threonine 68 – Cell Signaling, Danvers, MA, USA) was performed at 4 °C overnight. The following day the membranes were washed 5-times with TBS, each time for 5 minutes, and once with TBS, for 10 minutes, and then incubated with an appropriate secondary antibody (DakoCytomation, Glostrup, Denmark) for 1 h at room temperature. Band detection was performed using a chemiluminiscence detection kit (Roche, Basel, Switzerland). To ensure equal protein loading, each membrane was reprobed and β-actin was detected. At least three independent protein evaluations were performed.

### Statistical analysis

The descriptive statistics of the results were calculated in Microsoft Office Excel 2010 (Microsoft, Redmond, WA, USA) or GraphPad Prism 5 biostatistics (GraphPad Software, La Jolla, CA, USA). For quantitative data, normality testing was performed to assess whether parametric or nonparametric tests should be used. The statistics for comet assay results were computed using NCSS software (NCSS, Kaysville, UT, USA). Differences within the groups were evaluated using the nonparametric Kruskal–Wallis one-way analysis of variance, followed by Dunn’s post-hoc test with Bonferroni’s modification. All experimental data were expressed as median and 25th and 75th percentiles. For other cell culture experiments all the values were expressed as arithmetic means with S.D. of triplicates. For experiments with parametric variables, the significant differences between the groups were analysed using the Student’s t-test.

### Calculation of IC_50_ values

For IC_50_ values calculations data from viability determined by use of XTT assay were processed by using GraphPad Prism 5 biostatistics (GraphPad Software, USA) software. Drug concentrations were plotted against cell viability and the IC_50_ values were determined using non-linear regression.

## Electronic supplementary material

Supplementary Information
