# Supplementary material for: Scoulerine affects microtubule structure, inhibits proliferation, arrests cell cycle and thus culminates in the apoptotic death of cancer cells
Source: Sci Rep. 2018 Mar 19;8:4829. doi: 10.1038/s41598-018-22862-0 (PMC5859271; doi:10.1038/s41598-018-22862-0)
Supplement: Supplementary file 1 — Supplementary Information [file 41598_2018_22862_MOESM1_ESM.doc]

**Supplementary Material**

**Scoulerine affects microtubule structure, inhibits proliferation, arrests cell cycle and thus culminates in the apoptotic death of cancer cells**

Klara Habartova^1^, Radim Havelek^1,*^, Martina Seifrtova^1^, Karel Kralovec^2^, Lucie Cahlikova^3^, Jakub Chlebek^3^, Eva Cermakova^4^, Nadezda Mazankova^1^, Jana Marikova^5^, Jiri Kunes^5^, Lucie Novakova^6^, Martina Rezacova^1^

^1^Department of Medical Biochemistry, Faculty of Medicine in Hradec Kralove, Charles University, Simkova 870, Hradec Kralove 500 03, Czech Republic

^2^Department of Biological and Biochemical Sciences, Faculty of Chemical Technology, University of Pardubice, Studentska 573, Pardubice 532 10, Czech Republic

^3^ADINACO Research group, Department of Pharmaceutical Botany and Ecology, Faculty of Pharmacy, Charles University, Heyrovskeho 1203, Hradec Kralove 500 05, Czech Republic

^4^Department of Medical Biophysics, Faculty of Medicine in Hradec Kralove, Charles University, Simkova 870, Hradec Kralove 500 03, Czech Republic

^5^Department of Inorganic and Organic Chemistry, Faculty of Pharmacy, Charles University, Heyrovskeho 1203, Hradec Kralove 500 05, Czech Republic

^6^Department of Analytical Chemistry, Faculty of Pharmacy, Charles University, Heyrovskeho 1203, Hradec Kralove 500 05, Czech Republic

*Corresponding author

Radim Havelek, Ph.D.

Department of Medical Biochemistry,

Faculty of Medicine in Hradec Kralove,

Charles University,

Simkova 870,

500 03 Hradec Kralove,

Tel.: +420495816293,

E-mail address: [havelekr@lfhk.cuni.cz](mailto:havelekr@lfhk.cuni.cz)


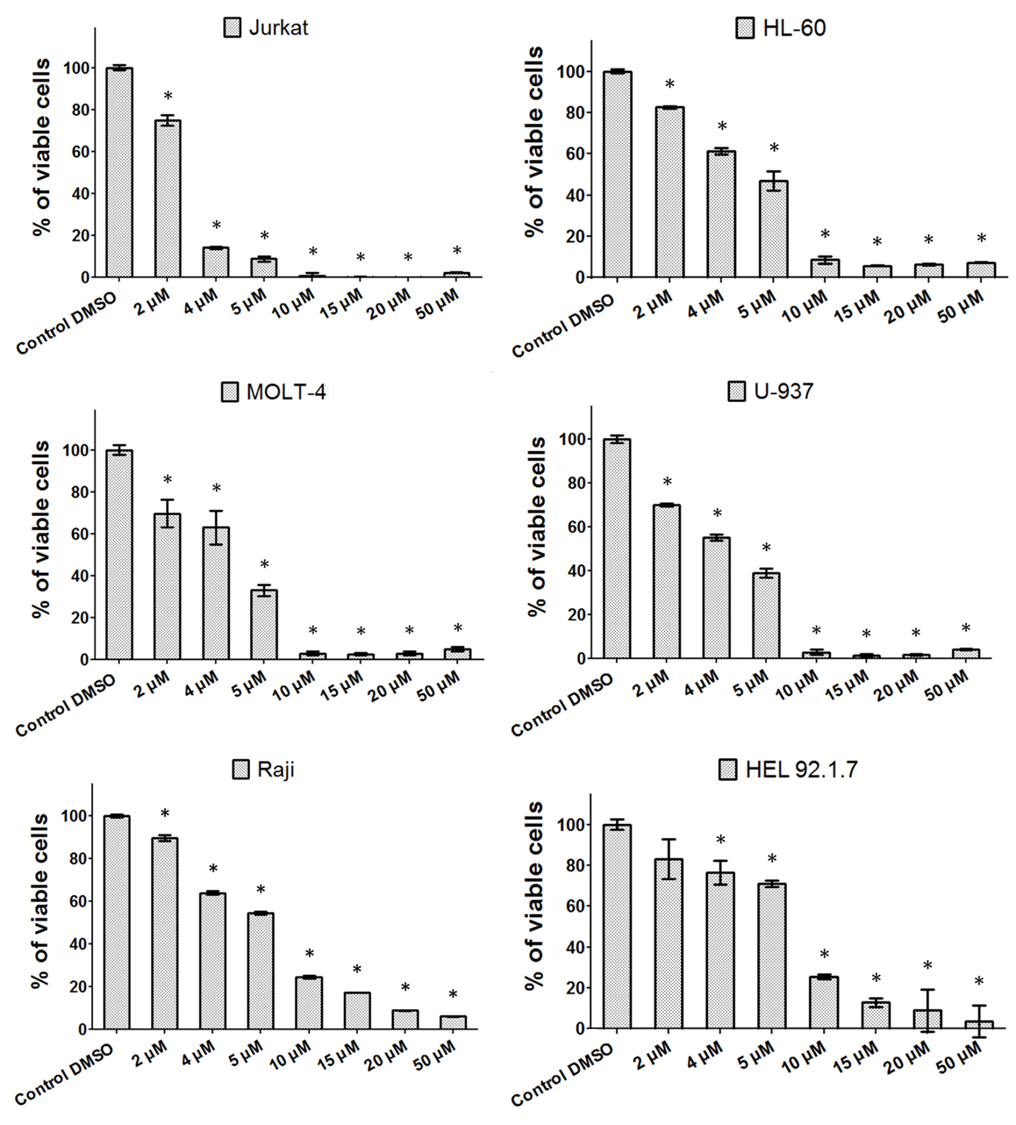


**Supplementary Fig. 1.** Cytotoxicity of scoulerine. Cell proliferation and viability of Jurkat, MOLT-4, Raji, HL-60, U-937 and HEL 92.1.7 cells measured by using XTT assay 48 h after treatment. Viability is referred to cells treated with 0.1% DMSO (Control DMSO). Data are shown as mean values ± SD of at least three independent experiments. * - significantly different to control (P ≤ 0.05).

**
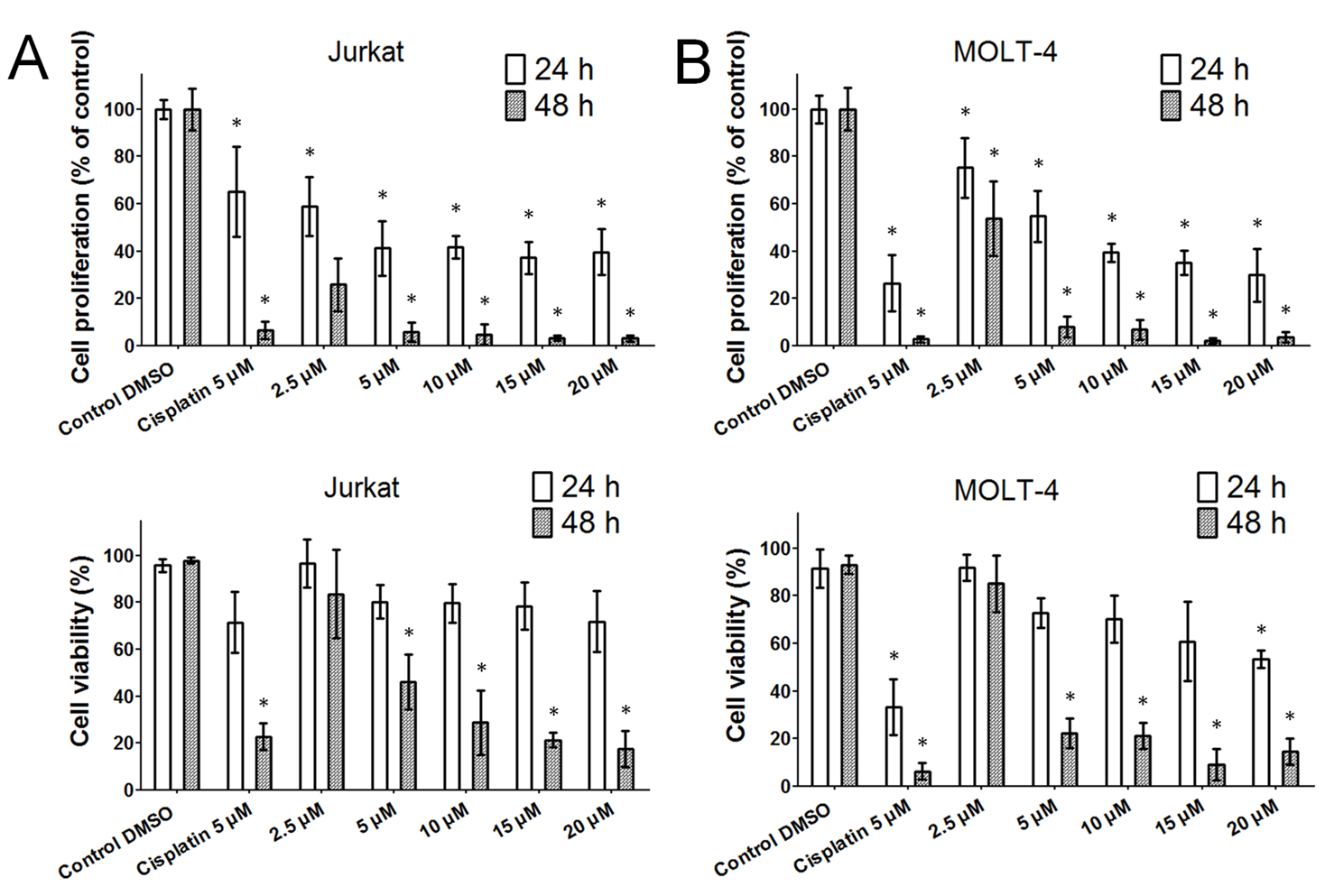
**

**Supplementary Fig. 2.** The effect of scoulerine on the proliferation and viability of Jurkat (A) and MOLT-4 (B) leukemic cells. Changes in the proliferation and viability were monitored by Trypan blue exclusion test for 24 and 48 h of treatment. Results are shown as mean ± SD from three experiments. * - significantly different to control (P ≤ 0.05). Cells treated with 5 µM cisplatin were used as positive control.

**
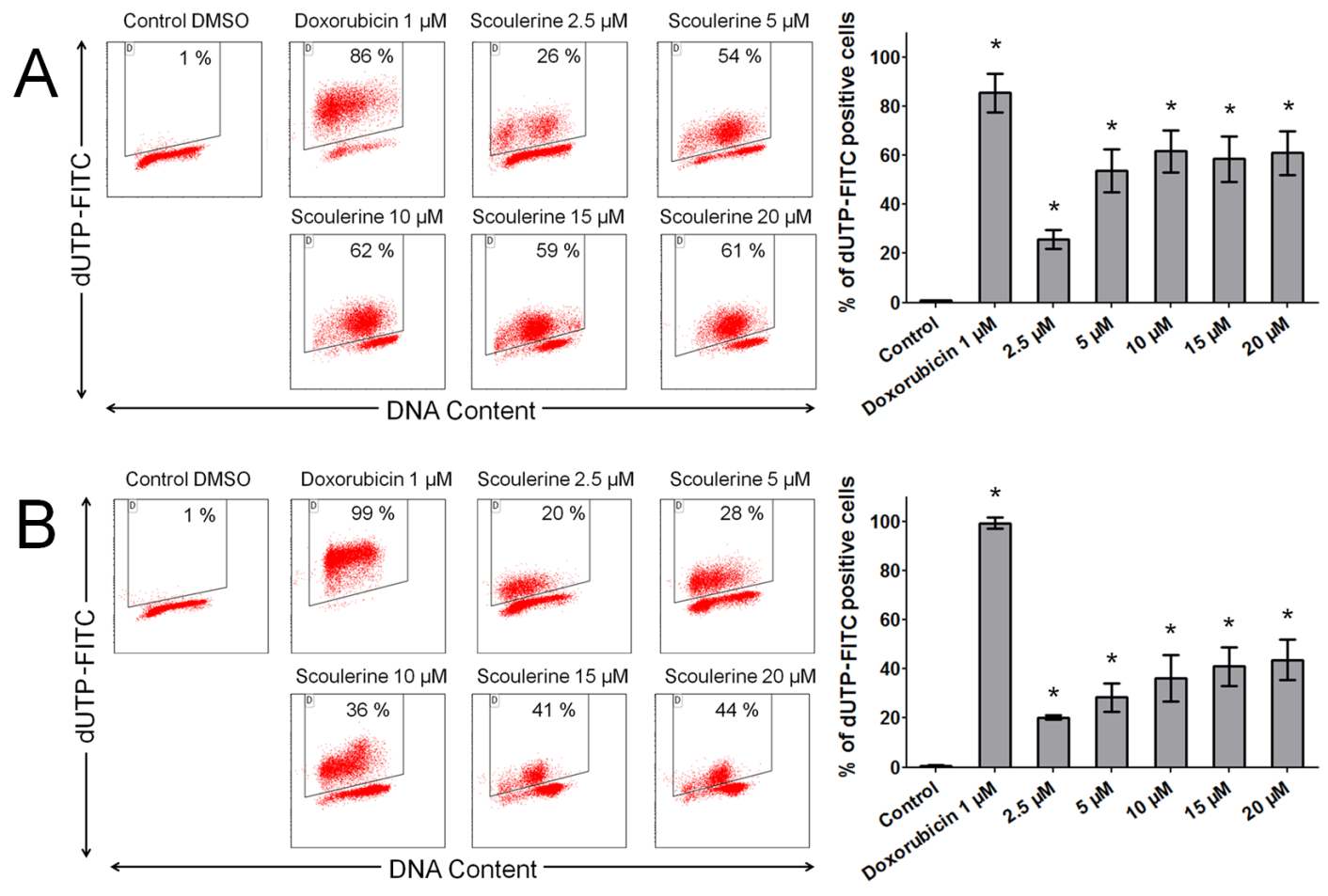
**

**Supplementary Fig. 3.** The effect of scoulerine on the DNA fragmentation in Jurkat (A) and MOLT-4 (B) leukemic cells 24 h after the treatment. The quantitative data from flow cytometry are presented as histograms which show the percentage of cells with a DNA strand breaks terminal deoxynucleotidyl transferase-mediated FITC-dUTP nick end-labelling. Representative dot plots of one of three independent measurements are shown. Cells treated with 1 µM doxorubicin, a topoisomerase II inhibitor, were used as positive control. The bar graph displays the percentage of cells that are TUNEL positive (mean ± SD, n = 3). * - significantly different to control (P ≤ 0.05).

***
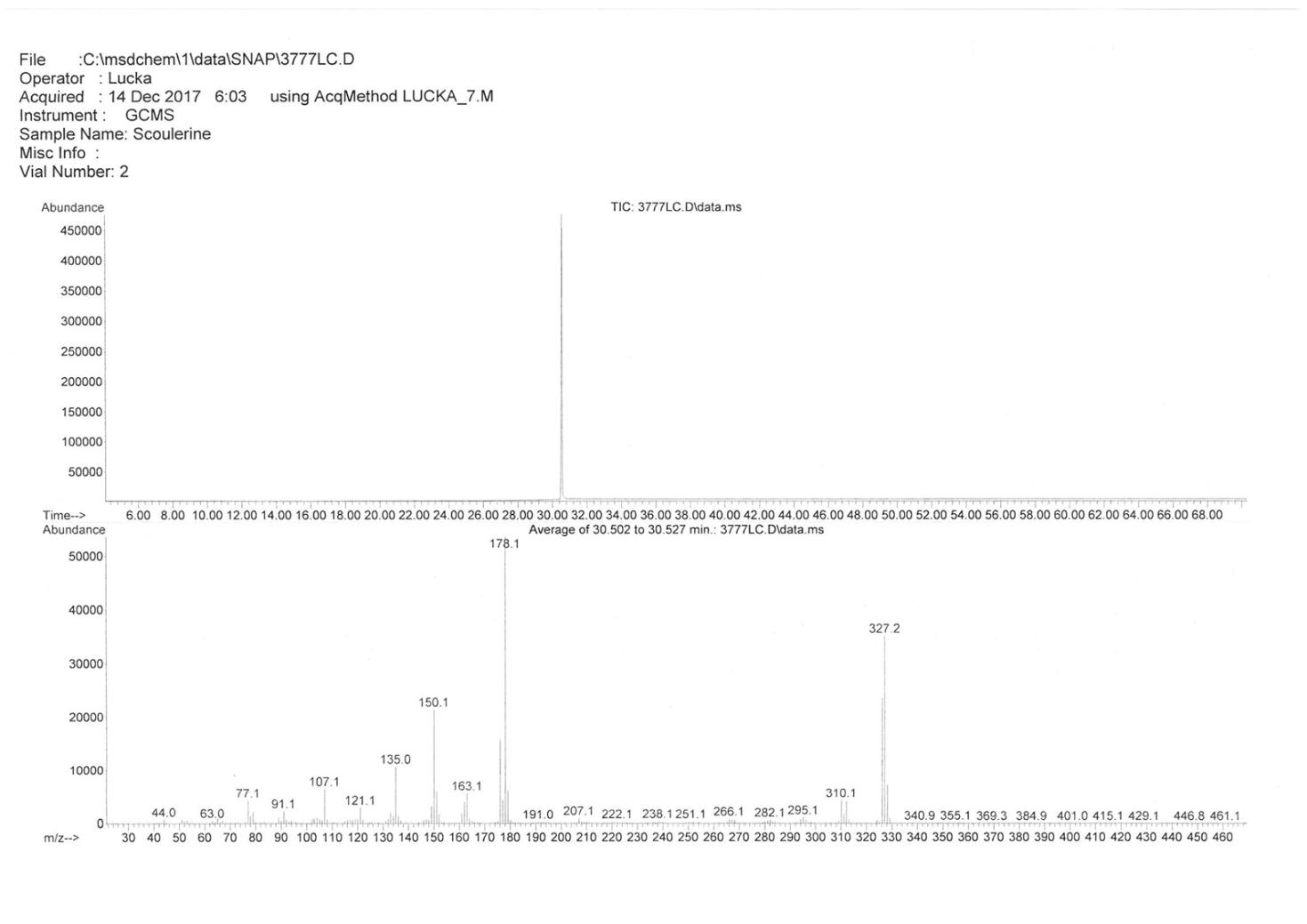
***

**Supplementary Fig. 4.** GC/MS analysis of scoulerine **(1)**

**Supplementary Fig. 5.** HPLC analysis of scoulerine **(1)**

**
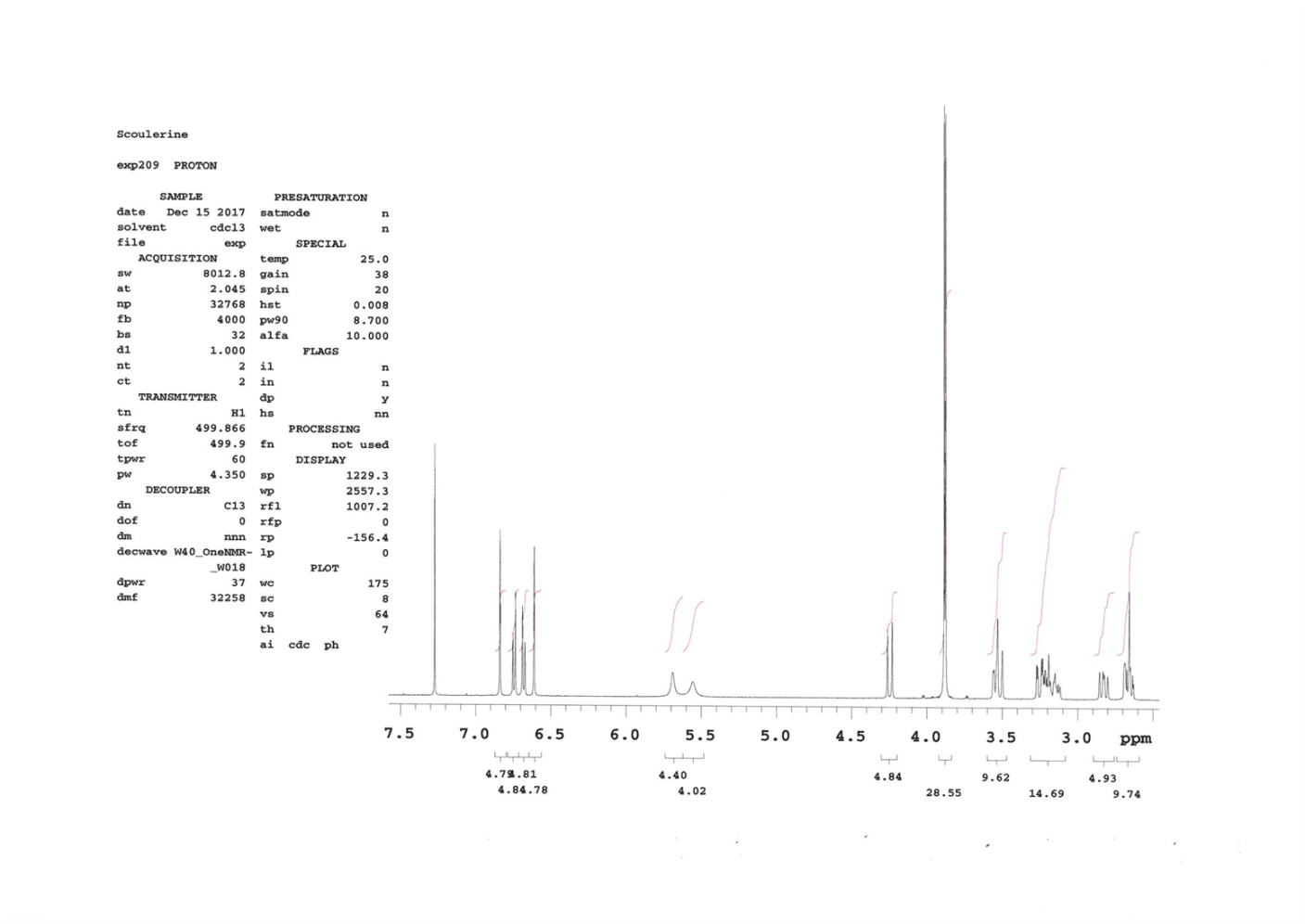
**

**Supplementary Fig. 6.** ^1^H-NMR analysis of scoulerine **(1)**

**
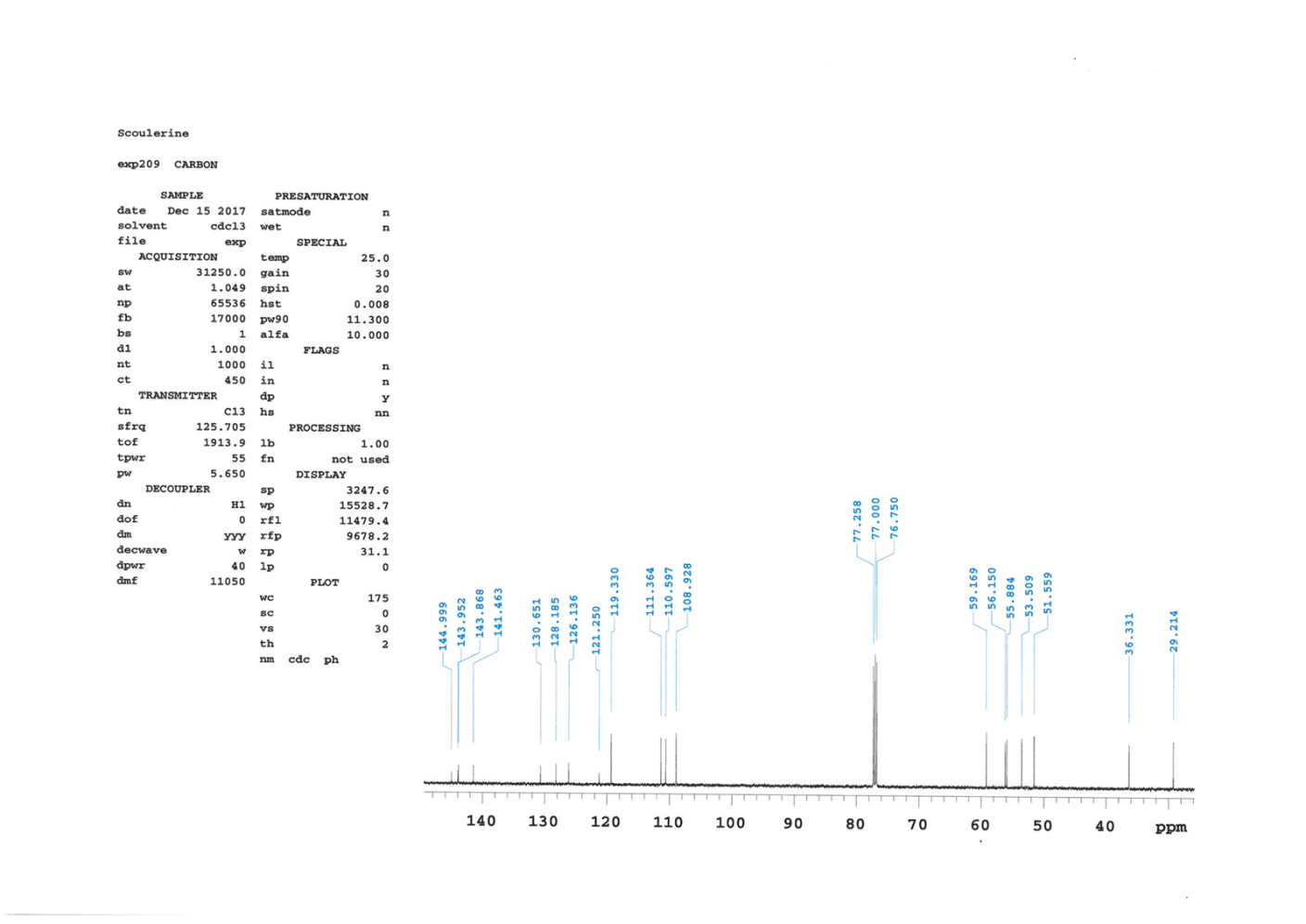
**

**Supplementary Fig. 7.** ^13^C-NMR analysis of scoulerine **(1)**

**
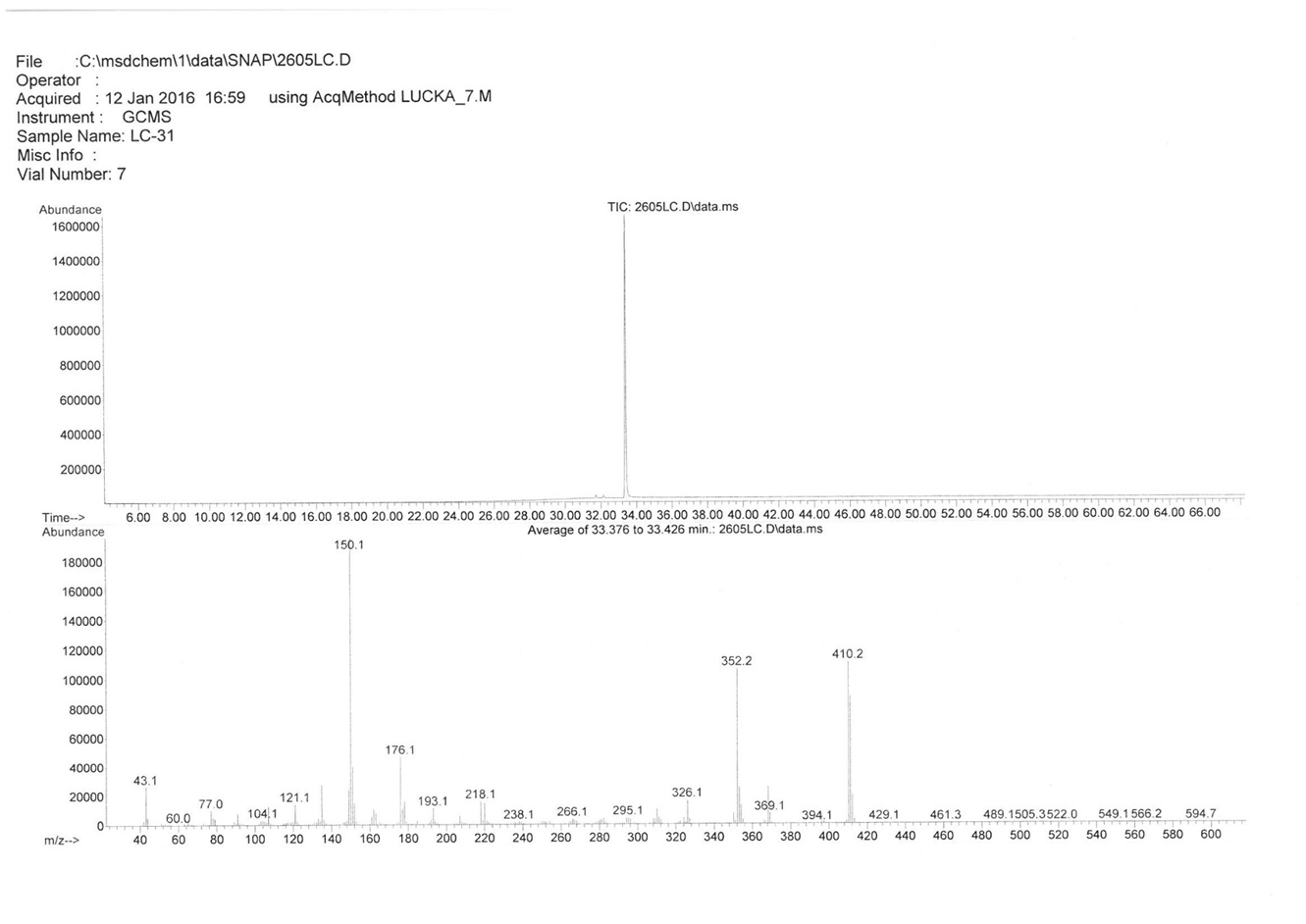
**

**Supplementary Fig. 8.** GC/MS analysis of 2,9-Di-*O*-Acetylscoulerine (**2**)

|  | **Name** | **Retention Time** | **Area** | **% Area** |
| --- | --- | --- | --- | --- |
| **1** |  | **2.015** | **1807** | **1.59** |
| **2** |  | **2.388** | **107837** | **95.04** |
| **3** |  | **2.473** | **3817** | **3.36** |

**Supplementary Fig. 9.** HPLC analysis of 2,9-Di-*O*-Acetylscoulerine (**2**)

**
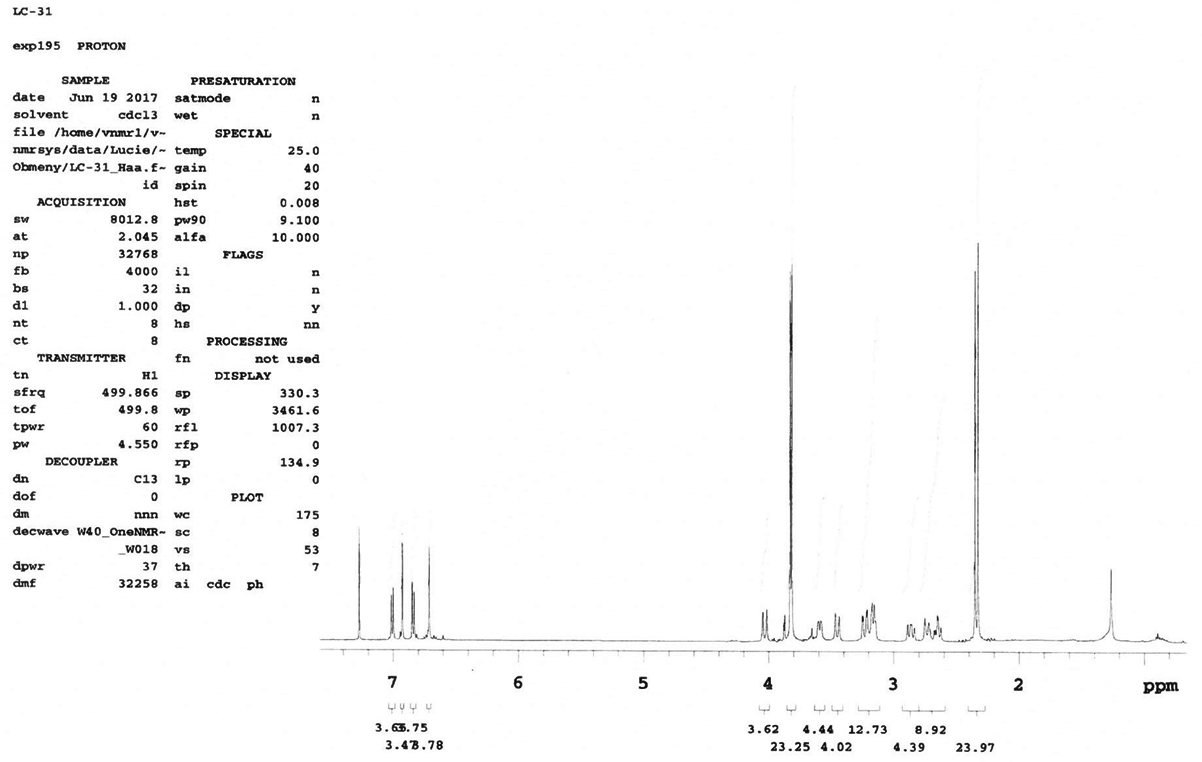
**

**Supplementary Fig. 10.** ^1^H-NMR analysis of 2,9-Di-*O*-Acetylscoulerine (**2**)

***
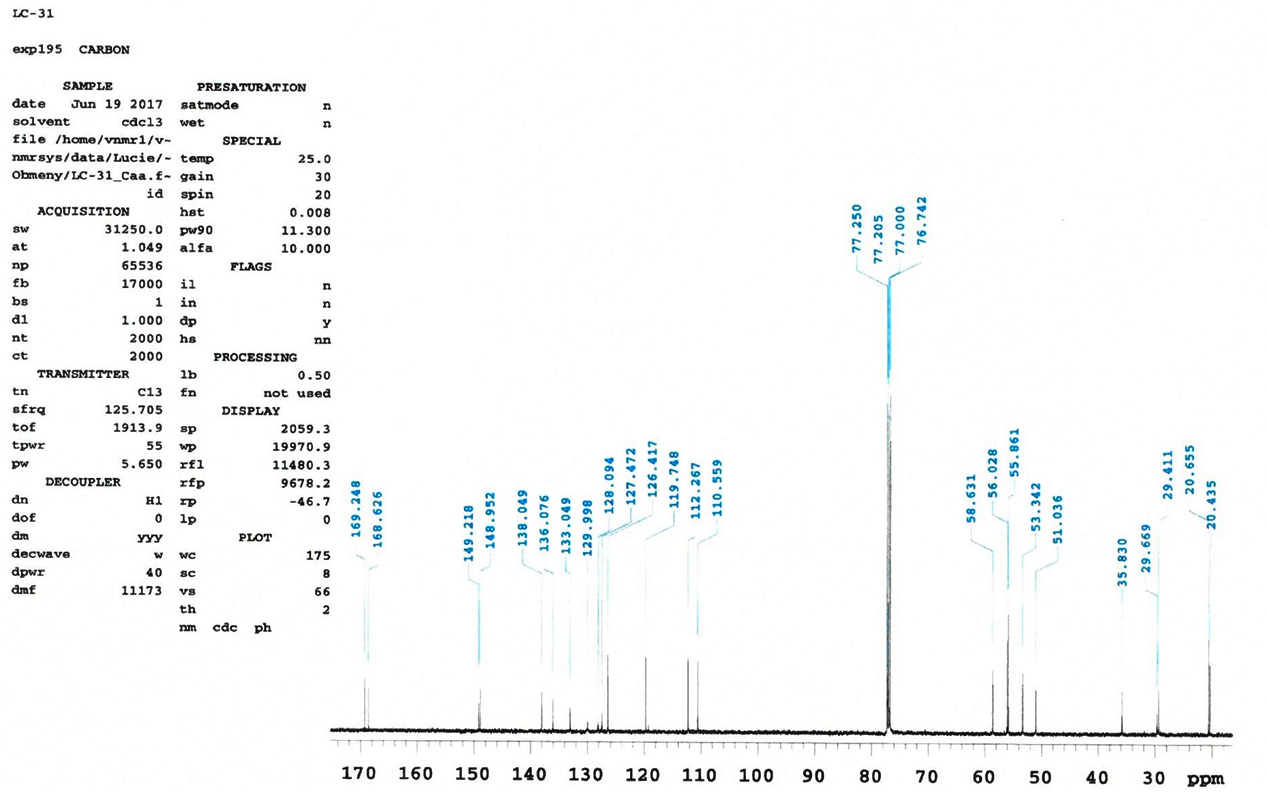
***

**Supplementary Fig. 11.** ^13^C-NMR analysis of 2,9-Di-*O*-Acetylscoulerine (**2**)

**
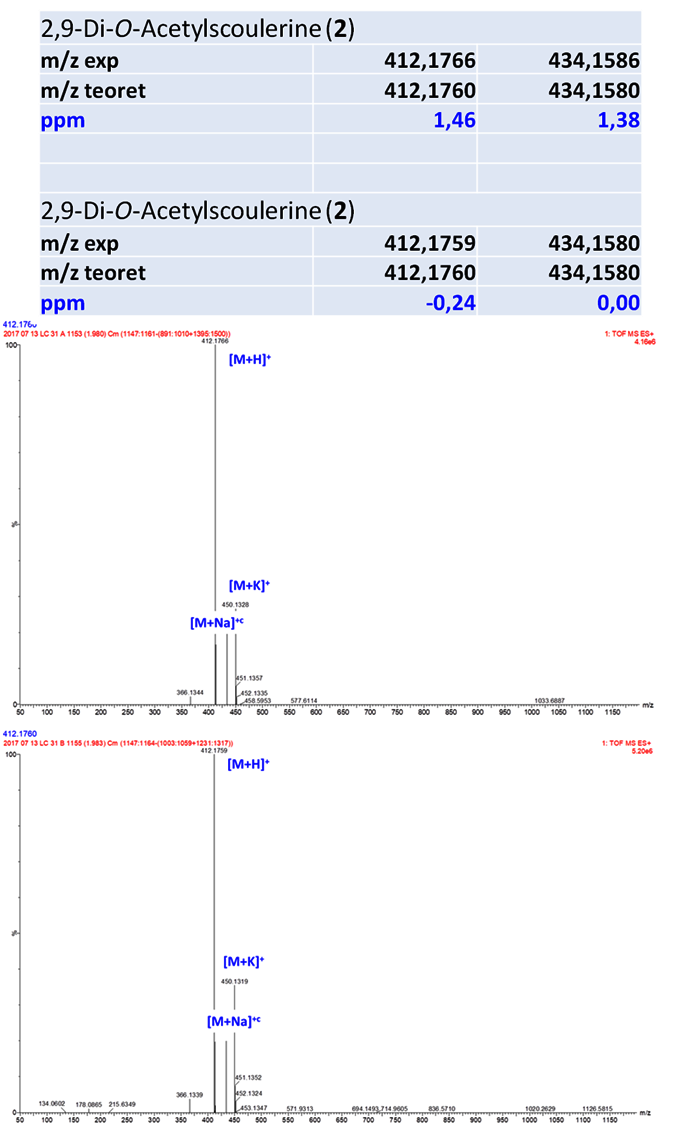
**

**Supplementary Fig. 12.** HRMS analysis of 2,9-Di-*O*-Acetylscoulerine (**2**)

**
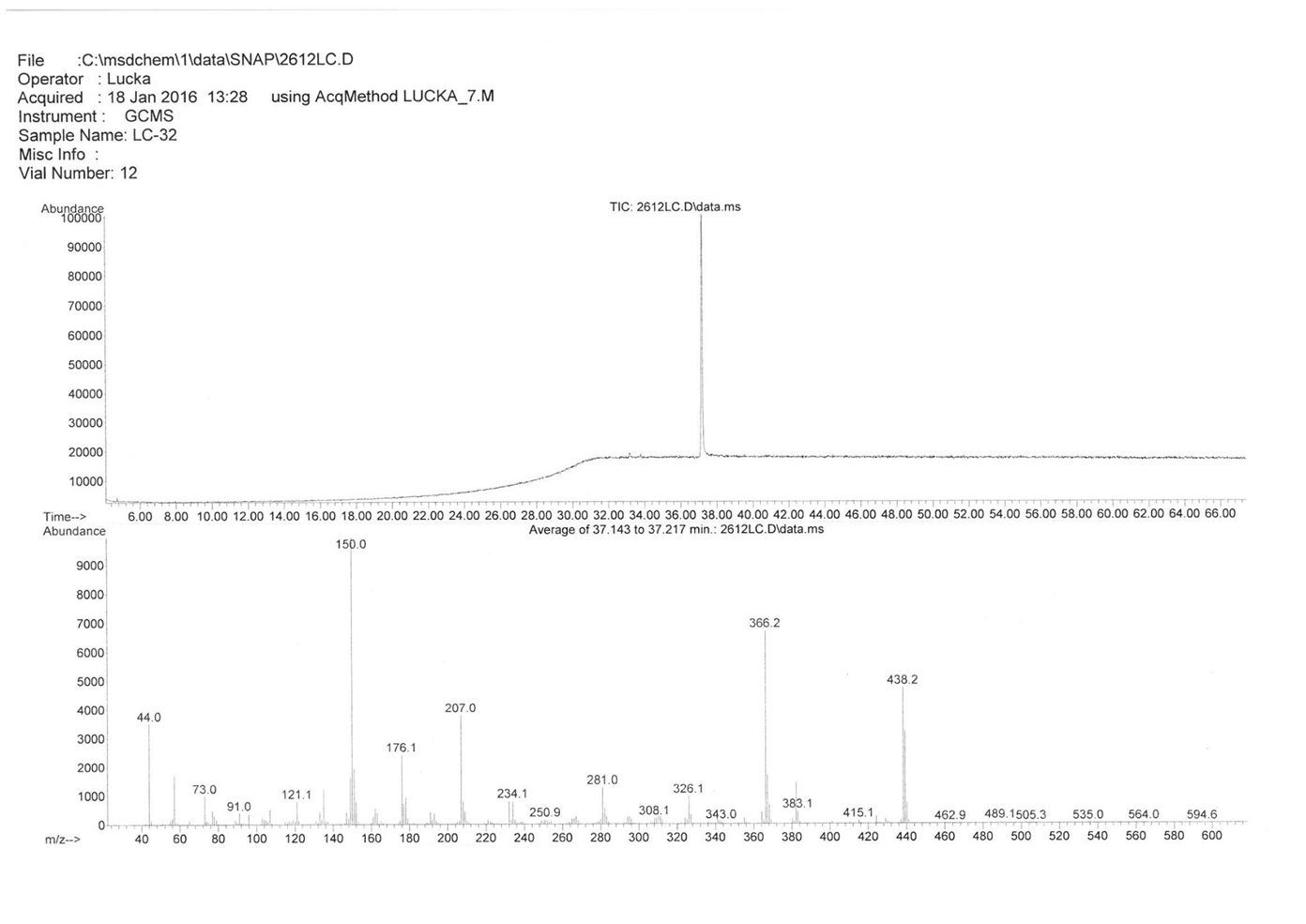
**

**Supplementary Fig. 13.** GC/MS analysis of 2,9-Di-*O*-Propionylscoulerine **(3)**

|  | **Name** | **Retention Time** | **Area** | **% Area** |
| --- | --- | --- | --- | --- |
| **1** |  | **2.242** | **6524** | **1.59** |
| **2** |  | **2.555** | **2900** | **0.71** |
| **3** |  | **2.651** | **6805** | **1.66** |
| **4** |  | **2.781** | **393872** | **96.04** |
|  |  |  |  |  |

**Supplementary Fig. 14.** HPLC analysis of 2,9-Di-*O*-Propionylscoulerine **(3)**

**
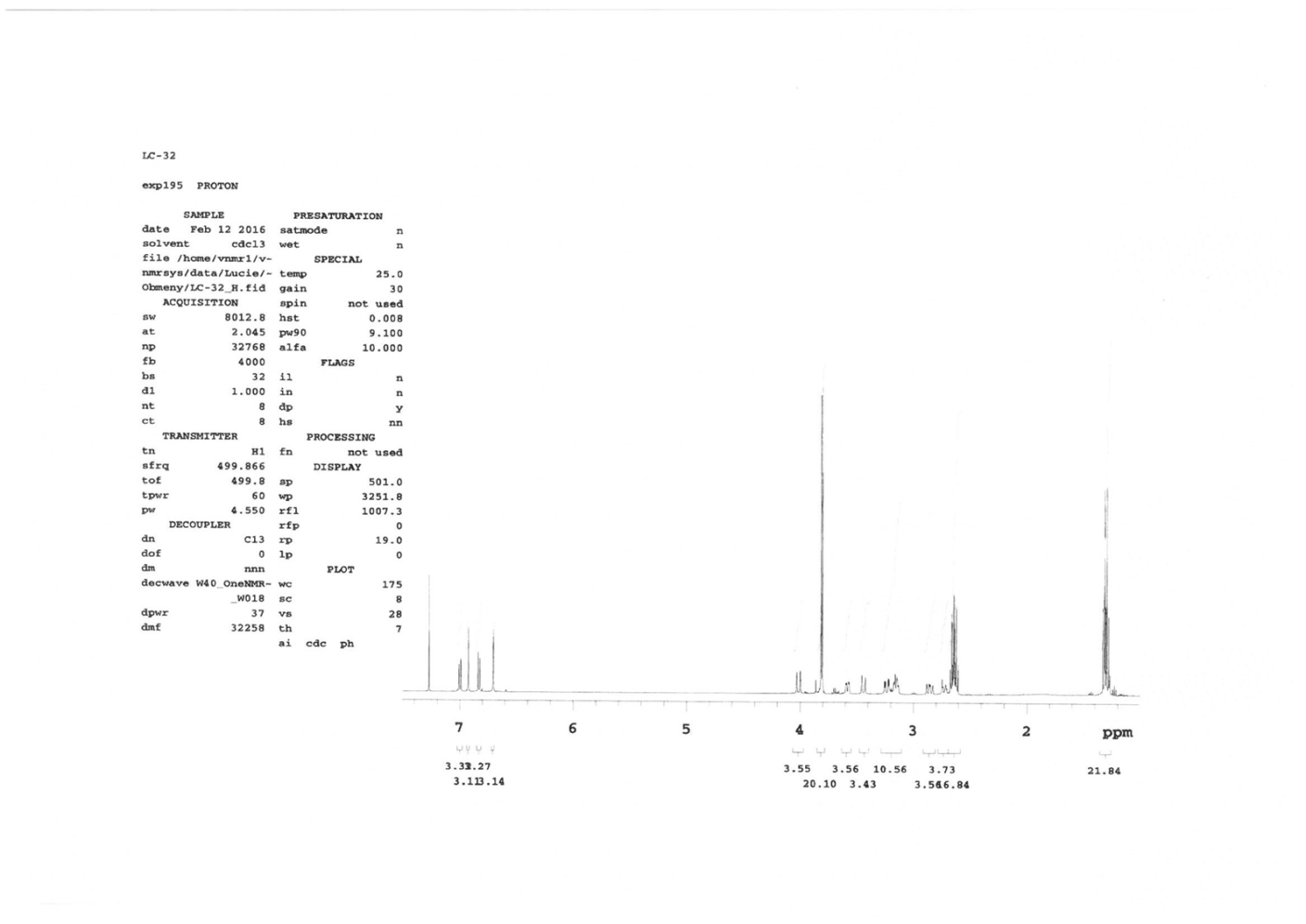
Supplementary Fig. 15.** ^1^H-NMR analysis of 2,9-Di-*O*-Propionylscoulerine **(3)**

**
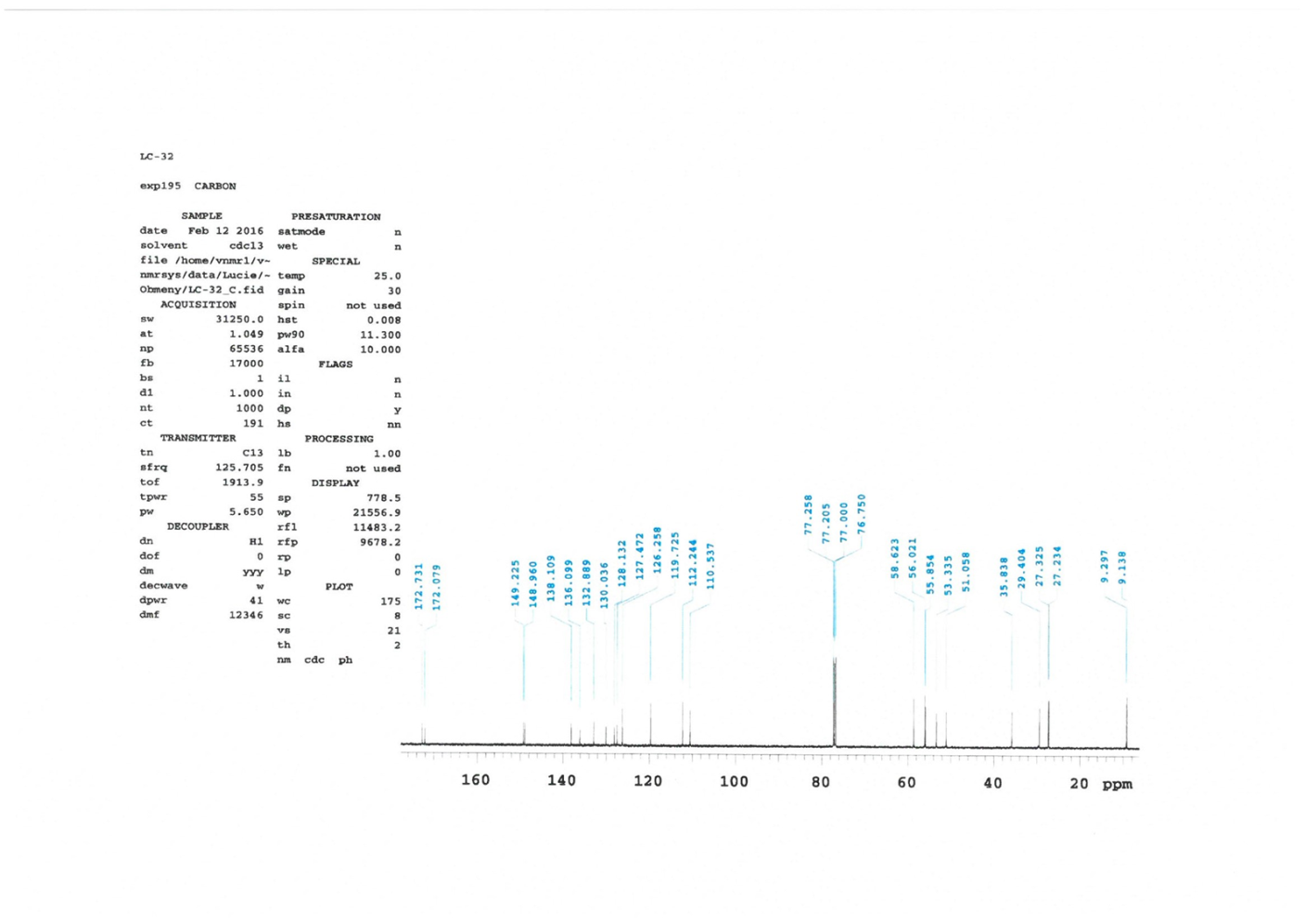
**

**Supplementary Fig. 16.** ^13^C-NMR analysis of 2,9-Di-*O*-Propionylscoulerine **(3)**

**
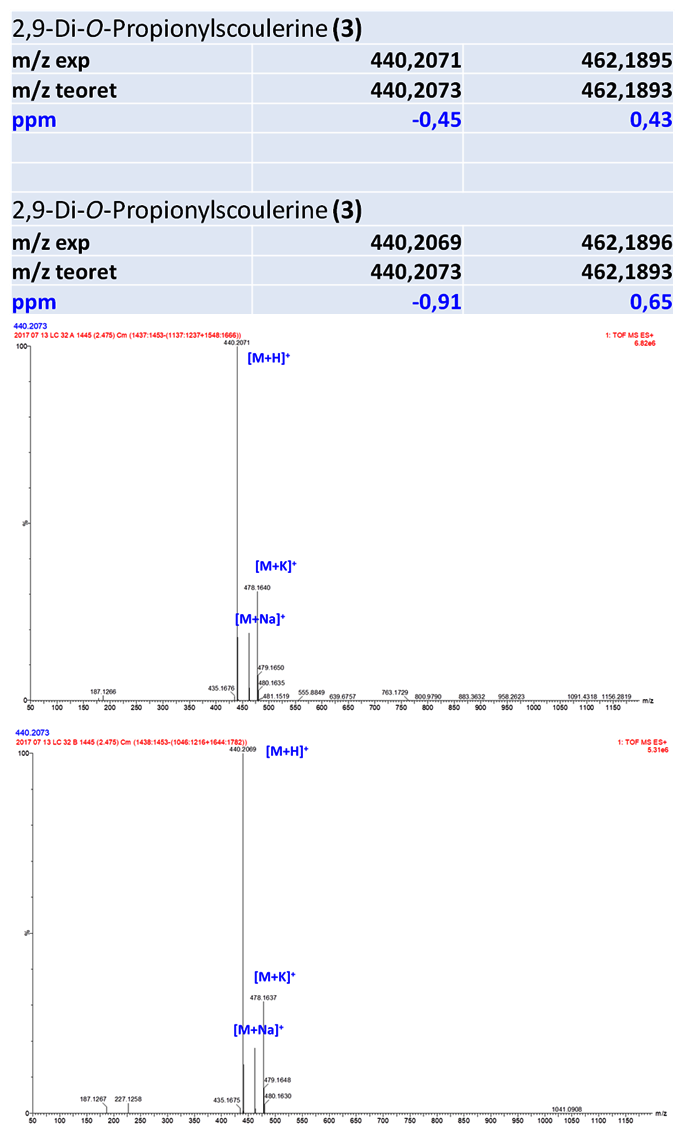
**

**Supplementary Fig. 17.** HRMS analysis of 2,9-Di-*O*-Propionylscoulerine **(3)**

**
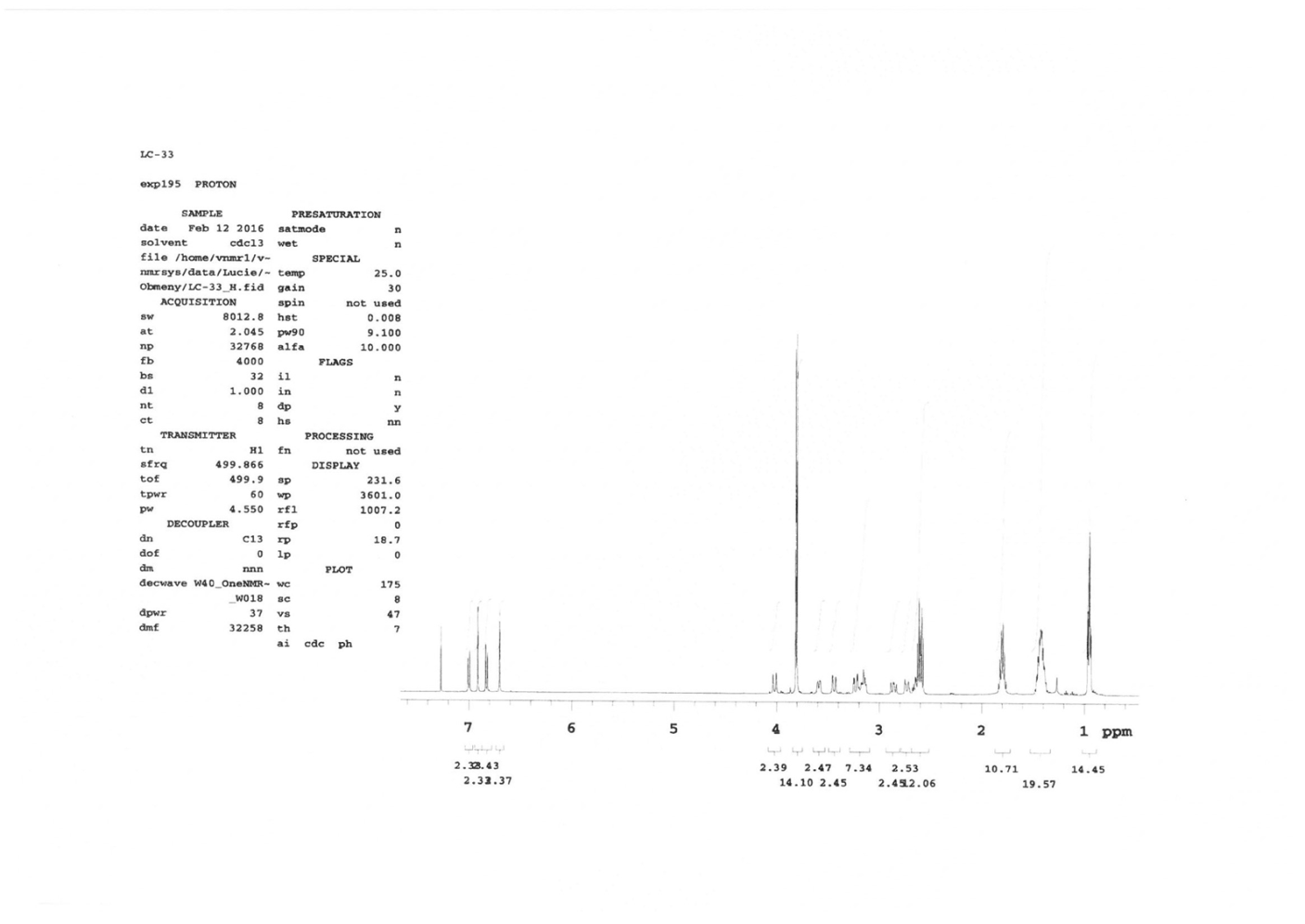
**

**Supplementary Fig. 18.** ^1^H-NMR analysis of 2,9-Di-*O*-Hexanoylscoulerine **(4)**

|  | **Name** | **Retention Time** | **Area** | **% Area** |
| --- | --- | --- | --- | --- |
| **1** |  | **3.014** | **3335** | **0.74** |
| **2** |  | **3.388** | **4490** | **0.99** |
| **3** |  | **3.915** | **444118** | **98.27** |

**Supplementary Fig. 19.** HPLC analysis of 2,9-Di-*O*-Hexanoylscoulerine **(4)**

**
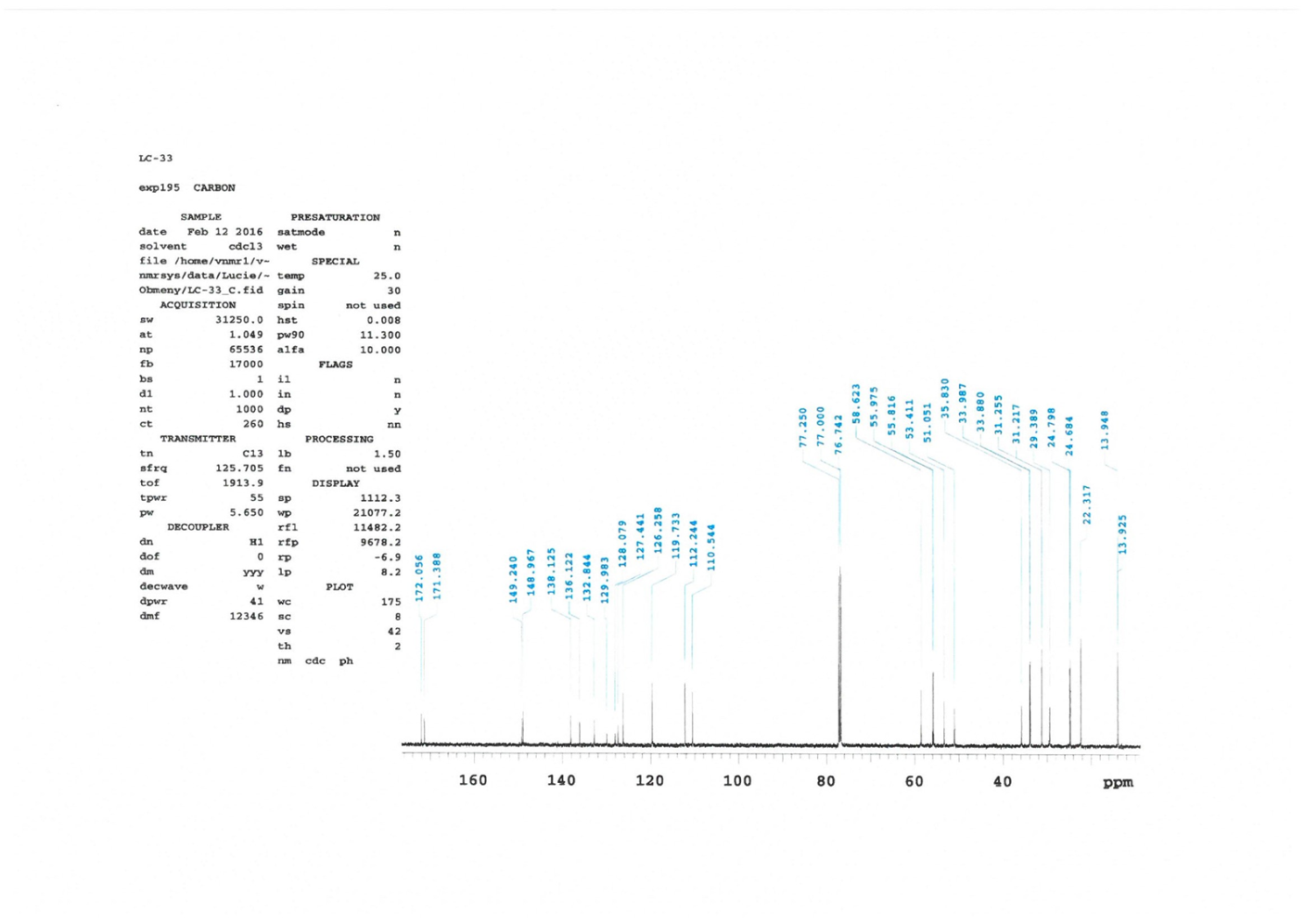
Supplementary Fig. 20.** ^13^C-NMR analysis of 2,9-Di-*O*-Hexanoylscoulerine **(4)**

**
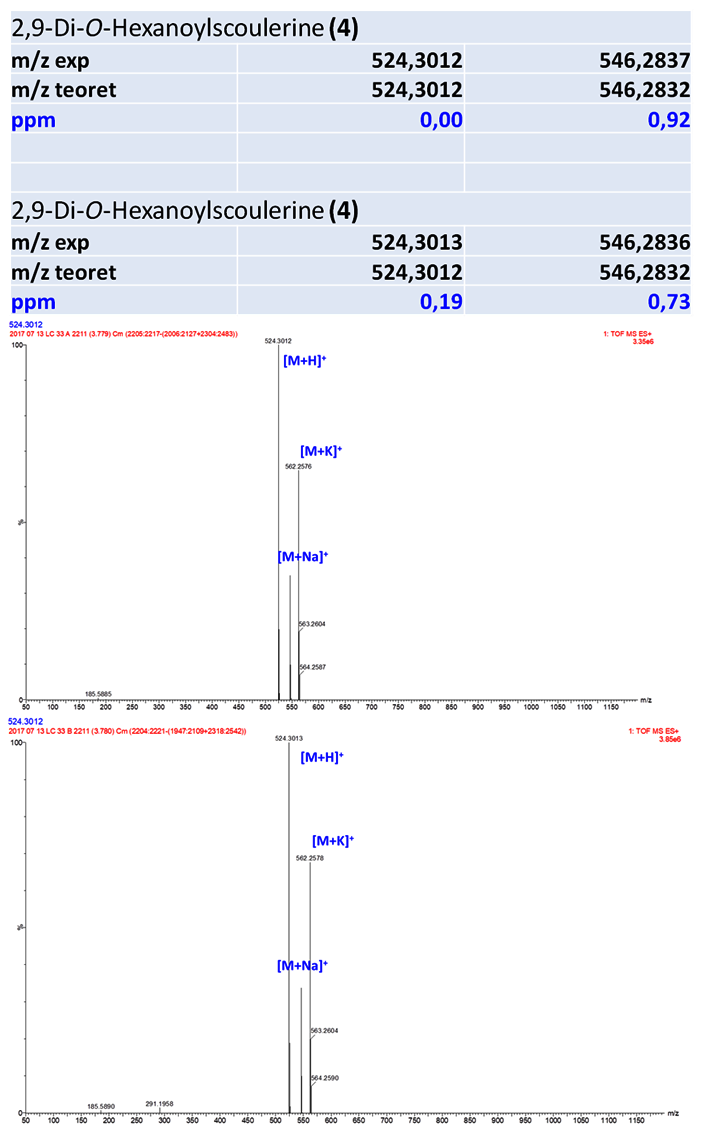
**

**Supplementary Fig. 21.** HRMS analysis of 2,9-Di-*O*-Hexanoylscoulerine **(4)**
